# Functional protein nanostructures: a chemical toolbox

**DOI:** 10.1039/c8cs00590g

**Published:** 2018-11-19

**Authors:** Seah Ling Kuan, Fernando R. G. Bergamini, Tanja Weil

**Affiliations:** a Max-Planck Institute for Polymer Research , Ackermannweg 10 , 55128 Mainz , Germany . Email: weil@mpip-mainz.mpg.de ; Email: kuan@mpip-mainz.mpg.de; b Institute of Inorganic Chemistry I – Ulm University , Albert-Einstein-Allee 11 , 89081 Ulm , Germany; c Institute of Chemistry , Federal University of Uberlândia – UFU , 38400-902 Uberlândia , MG , Brazil

## Abstract

Nature has evolved an optimal synthetic factory in the form of translational and posttranslational processes by which millions of proteins with defined primary sequences and 3D structures can be built. Nature's toolkit gives rise to protein building blocks, which dictates their spatial arrangement to form functional protein nanostructures that serve a myriad of functions in cells, ranging from biocatalysis, formation of structural networks, and regulation of biochemical processes, to sensing. With the advent of chemical tools for site-selective protein modifications and recombinant engineering, there is a rapid development to develop and apply synthetic methods for creating structurally defined, functional protein nanostructures for a broad range of applications in the fields of catalysis, materials and biomedical sciences. In this review, design principles and structural features for achieving and characterizing functional protein nanostructures by synthetic approaches are summarized. The synthetic customization of protein building blocks, the design and introduction of recognition units and linkers and subsequent assembly into structurally defined protein architectures are discussed herein. Key examples of these supramolecular protein nanostructures, their unique functions and resultant impact for biomedical applications are highlighted.

## Abstract 2

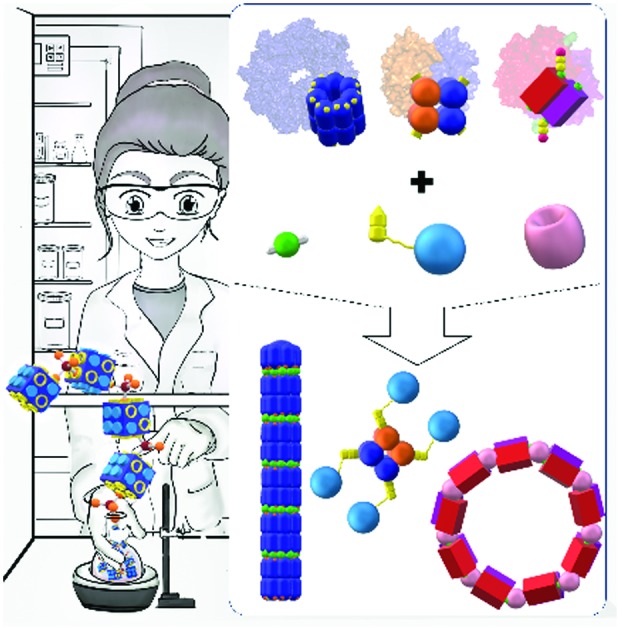
Functional protein nanostructures hold immense potential for a broad range of applications, *e.g.*, in material and biomedical sciences. In this article, the development of chemical toolboxes to build precise functional protein nanostructures that go beyond Nature's portfolio and their applications are summarized.

## 1. Introduction

Protein nanostructures (PNs) are ubiquitous in Nature and fuel the complex cellular machinery through provision of functions, structural frameworks and molecular recognition. These biomacromolecules are in the first instance prepared in a sequence defined manner through transcription and translation processes, which lead to secondary and tertiary structures that confer functions such as catalytic activity.^1^ More complex arrangements such as oligomers, polymers and networks can also be created through protein–protein interactions.^2^ In this manner, Nature has evolved its own optimized toolbox through millennia of evolution that allows a plethora of PNs to be constructed. Essentially, such unique functional PNs are formed through precise molecular interactions of the monomeric protein units. For example, viral capsids consist of multiple copies of a monomeric protein unit through non-covalent interactions, resulting in the formation of stable polyhedral structures that are essential for protecting, storing, and transporting genetic information.^3^ Another example are the highly potent bacterial exotoxins, such as botulinum toxins, which consist of enzymatic, translocation, and cell binding domains. These discrete structural domains serve individual functions that, in combination, give rise to one of Nature's most potent weaponry. But additional functional and structural diversities can also be conferred to protein building blocks (PBs) through post-translational modifications to expand the repertoire of functional and dynamic nanostructures.^4^,^5^

Inspired by Nature's machinery, there has been an emergence of research activities to evolve synthetic strategies that allow the rational design to construct functional PNs. These toolkits have shown great prospects in terms of preparation of PNs for different fields such as catalysis, biotechnology, and biomedicine.^6^–^9^ In particular, such well-defined nanostructures, which possess bioactivity and attractive materials properties, will be highly relevant for biomedical applications given the stringent demands for stability, biocompatibility, and biosafety. However, the field is fraught with challenges largely due to the complexity of protein surfaces. The exact spatial arrangement of proteins requires strict control of directionality, but the orientation of molecular recognition entities on protein surfaces can often not be predicted from the onset.^6^ With the progress in bioinformatics, biotechnology, chemical biology, and analytical tools, there has been an increased understanding in protein structures, folding, and protein–protein interactions.^10^ In terms of applications, mild conditions are required to preserve the activity of the protein components in the complexes. Therefore, non-covalent or dynamic covalent strategies have emerged as valuable synthesis tools to impart molecular recognition units since they are reversible and should have less impact on the tertiary structure and activity of the protein components.^11^–^13^ In fact, most protein assemblies found in Nature are formed by non-covalent interactions, which allow for the rapid protein assembly and disassembly, responding and reporting to changes in their local physiological environments, such as variations in pH or ionic gradient, ligand concentrations or light.^2^ In contrast, chemical crosslinking is limited by the stability of proteins to the reaction conditions, and dynamic features are mostly lost during such processes.

To date, the utilization of biotechnological tools for engineering entirely new protein nanostructures with desired functional features has met with some notable success. For instance, the assembly of protein nanostructures through genetic engineering was reported, whereby natural oligomerizing protein domains were fused together through a rigid, peptide linker to form a defined cage-like structures such as the 12-mer tetrahedral cage.^11^,^14^ Coiled–coiled peptides have also been used to induce protein dimerization as in split luciferase reporters,^15^ and *de novo* design has been adopted on small modular domains to form distinct 3D structures as in tetrahedral nanocages.^16^ However, in some of the designs, greater predictability of the resultant PNs is required, and the introduction of entirely new functions is still challenging. In addition, genetic engineering has limitations if synthetic entities such as dyes need to be introduced, *e.g.* to further expand Nature's functional portfolio. Likewise, protein aggregation and laborious protein purification are further drawbacks of genetic engineering. Chemical modifications of proteins were considered less attractive for the preparation of protein building blocks due to lack of controlling of the reaction sites. Nevertheless, the rapid progress in terms of site-selective chemical modifications in the last decade^17^,^18^ has stimulated important advances in the field. Moreover, since Nature uses a combination of genetic and chemical tools to achieve infinite possibilities, the merger of different contemporary strategies in the expanding engineering toolbox has been capitalized to prepare more complex macromolecular structures such as heterofunctional proteins like chemical fusion proteins^19^,^20^ or higher-order protein conjugates that have been applied for biological applications.^21^,^22^

There are a few recent reviews, which give a broad overview of protein assemblies through both biotechnological and chemical means.^23^,^24^ In this review, we focus on chemical approaches (toolboxes) to build precise PNs, with emphasis on the preparation of PBs, the design of supramolecular linkers (SLs) that guide self-assembly, stability of the resultant PNs and their applications that go beyond Nature's portfolio in functionality. “Simple” protein bioconjugates obtained solely by covalent crosslinking or fusion proteins expressed by recombinant engineering without any synthetic modification or without using any synthetic SLs are excluded and readers can refer to reviews elsewhere.^7^,^23^–^25^ First, the toolboxes and the essential components to prepare PBs required for the design and preparation of precisely defined nanostructures are summarized. This includes the individual PBs, namely, native, chemically modified and genetically engineered PBs, which are essential components for PN formation. In the next section, customized synthetic interconnecting conjugation reagents essential for controlled assembly of the PBs are highlighted, as well as their binding constants and stabilities. Such SLs control the spatial assembly of individual PBs to form the desired nanostructures. Characterization of the final PNs can be challenging and main techniques, together with the different PN morphologies, are introduced herein. In the last chapter, we summarize the functional PNs and highlight the perspective to solve urgent needs in biomedical applications.

## 2. Design principles of supramolecular protein nanostructures (PNs)

**Fig. 1:**
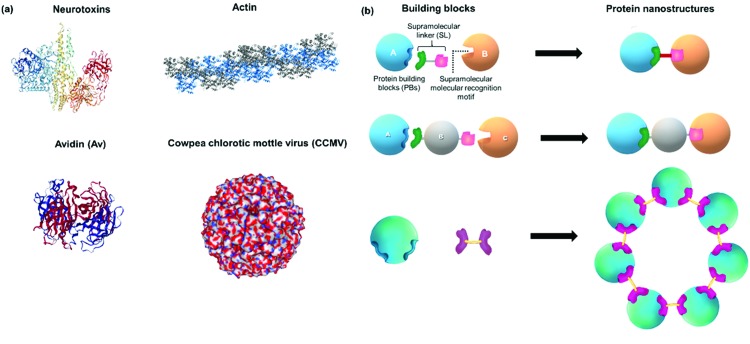
(a) Examples of protein nanostructures (PNs) in Nature: tripartite neurotoxin (PDB: 3BTA), tetrameric avidin (PDB: ; 1AVE), actin polymer and 3D virus capsids (PDB: ; 1CWP). (b) Schematic overview and design of distinct PNs formed by precise interactions of protein building blocks (PBs), supramolecular linkers (SLs) and supramolecular recognition motifs yielding *e.g.* dimeric, trimeric or ring-like nanostructures as examples. Protein images were created using the NGL viewer.^240^

Proteins are attractive building blocks for the design of functional nanomaterials due to their inherent bioactivity and multiple functionalities that provide a rich platform for inter- and intramolecular interactions. In order to form defined PNs, stringent control over directionality and spatial placement of the PBs is essential. Therefore, the surfaces of PBs should ideally be encoded with molecular information, *i.e.* contain the supramolecular recognition motifs, as anchor points that can interact with the respective SL in a “lock and key”-like mechanism for spontaneous generation of distinct, higher order protein assemblies (Fig. 1).^26^ For subsequent applications, it is a prerequisite that the PBs retain their structure and bioactivity during the modification and assembly processes. In the following, the design principles are discussed first highlighting (1) the selection of the respective native and modified PBs that contain the recognition motifs as hot spots. Subsequently, (2) the SLs interconnecting the PBs by complementary units that recognize the hotspots on the PBs in an orthogonal fashion are summarized to ultimately achieve (3) their controlled assembly into defined and functional PNs.

### 2.1. Selection of monomeric protein building blocks (PBs)

The geometry, functional groups, surface interactions, and ligand recognition of the PBs play an important role in the formation of defined PNs. Native PBs that already possess recognition motifs can be applied directly and represent the simplest of these essential components to form PB. In the case where native PBs are not available, chemical or genetic transformations will be required. In order to prepare protein dimers or homoprotein polymers, a single site of the protein is usually modified.^27^,^28^ In contrast, the formation of higher ordered structures such as rings or nanotubes requires the introduction of two or more recognition motif at the protein surface.^29^,^30^ The most straightforward strategy is the chemical modification of native proteins at already available single amino acid residues such as cysteines, disulfides, or amines. Otherwise, genetically engineered PBs have to be expressed if site-directed modification of the native protein is not possible. The modification sites for the introduction of recognition motifs are discussed. Table 1 summarizes some of the PBs reported in the literature for nanostructure formation and the introduced modifications.

**Table 1:** Summary PBs, type of modification, modification site and the recognition motifs

	Protein precursors	Function	Modification	Recognition motif
Native proteins	Lysozyme	Enzyme	None	Arg128
	Cytochrome *c*	Enzyme		Lys4 and Lys100
	Protamine	Nuclear protein		Positively charged surface amino acids
	(Strept)avidin	Tetrameric biotin-binding protein		Binding pockets of protein
	Concanavalin A, lectin A, soybean agglutinin	Tetrameric carbohydrate-binding protein		Binding pockets of protein
	Ferritin	Protein cage for iron storage		Surface charges
	Cowpea chlorotic mottle virus (CCMV)	Virus capsid		Surface charges
	Stable protein one (SP1)	Stress responsive protein		Positively charged surface amino acids and central cavity in protein
Chemically modified proteins	Human serum albumin	Blood plasma protein	Cys34	Biotin
	Somatostatin	Hormone	Cys3–Cys14	Biotin
	Insulin	Hormone	LysB29	Bipyridine
	Catalase	Hemeprotein	Lysine modification (statistical)	ssDNA (multiple)
Recombinant engineered proteins	Cytochrome *b*_562_	Hemeprotein	63Cys	Heme binding pocket
	Myoglobin	Hemeprotein	125Cys	Heme binding pocket
	C3 from *Clostridium botulinum*	Toxin enzyme	N-Terminal Cys	Biotin
	Cytochrome *cb*_562_	Hemeprotein	59Cys mutation at *i* and *i* + 4 positions: His59/63; His 73/77	1,10-Phenantroline histidine
	Alkaline phosphatase	Enzyme	N- or C-termini mutation	Biotin
	Split luciferase fragments	Enzyme fragments	N- or C-termini mutation	Phe-Gly-Gly
	Cyan or yellow fluorescent proteins (CFP, YFP)	Fluorescent protein	N- or C-termini mutation	Phe-Gly-Gly
	LiDPS protein cage	Ferritin protein cage	Cysteine mutation	Biotin
	Glutathione transferase dimer	Enzyme	N-Terminal polyhistidine	Polyhistidine
			N-Terminal	Phe-Gly-Gly
			137Cys	Phe-Gly-Gly
			137Cys, 138Cys	Histidine
	Chaperonin GroEL	Tetradecameric molecular chaperones	313Cys, 314Cys	Spiropyran

#### 2.1.1 Native PBs

Native PB assemblies form *via* specific interactions of protein surface patches or protein binding pockets. The interfacial area between interacting proteins plays an important role in protein–protein complexes, which are crucial for self-assembly processes in Nature.^31^ For instance, protein cages such as ferritin consist of multiple copies of a subunit assembled into highly defined 3D architectures and topologies. One common feature in these natural assemblies is the presence of charged amino acid residues that can interact *via* electrostatic forces on the subunit and assemble with the patchy surface subunits of oppositely charged residues to form defined supramolecular complexes.^32^,^33^

**Fig. 2:**
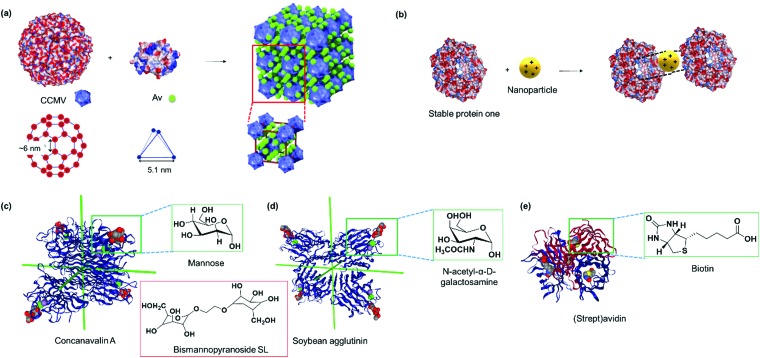
(a) The electrostatic surface of native cowpea chlorotic mottle virus and avidin (Av) with the location and geometry of the surface patches, which allow interactions to form binary protein crystals. Adapted with permission from ref. 34. Copyright 2014 Nature Publishing Group. (b) The electrostatic surface of stable protein one (SP1, PDB: ; 1TR0) and the central cavity that interacts with polycationic nanoparticles with specific dimensions. Blue denotes positive surface charges, red denotes negative surface charges, and white refer to neutral areas. (c) Tetrahedral concanavalin A (PDB: ; 1CVN) *versus* (d) slightly out of plane soybean agglutinin (PDB: ; 1SBF) that assembles with sugar units. Red inset in (c) shows a bismannopyranoside SL. Both PBs in (c and d) were viewed along the *D*_2_ symmetry axes for direct comparison. (e) (Strept)avidin tetramer (PDB: ; 1MK5) with the binding ligand biotin. Insets in (c–e) shows the chemical structure of the biotin ligand. Protein images were created using the NGL viewer.^240^

Kostiainen *et al.* devised an elegant strategy to self-assemble binary crystals from natural proteins comprising two different PBs with opposite charges that are presented as surface patches on the proteins.^34^ In this way, oppositely charged cowpea chlorotic mottle virus (CCMV) particles (isoelectric point (pI) of 3.8) and avidin (pI of 10.5) were assembled into binary crystals by using electrostatic interactions (Fig. 2a). Avidin offers the additional advantage that it can accommodate four biotin molecules facilitating the attachment of additional functionalities such as fluorescent dyes, enzymes or gold nanoparticles to impart a next degree of functions to the nanostructure.^34^ However, this method only allows the incorporation of PBs that meet the stringent requirements for electrostatic self-assembly and cannot be adopted to assemble two negatively charged PBs, such as CCMV with the green fluorescent protein with an isoelectric point (pI) of 5.

Stable protein one (SP1) forms a dodecameric ring-like protein with a diameter of approximately 11 nm, a central cavity of 2–3 nm, and a width of 4–5 nm.^35^ The acidic amino acids are mainly present at the top and bottom surface of the dodecamer SP1, and its unique topology and structural features have been exploited to control the directionality of electrostatic-induced self-assembly (Fig. 2b). Specifically, the symmetric concave of SP1 can accommodate globular nanoparticles in the centre of the double-layered nanoring to mitigate unidirectional growth.^35^ This method offers the advantage of assembling functional nanoparticles, which could be of interest for numerous applications such as light harvesting antennas.^35^

Overall, the electrostatic assembly based on protein surface topology offers simplicity as native PBs are used without the need for recombinant engineering or chemical modifications, and additional functional features have been introduced into the protein complexes as well. However, the charge distribution at protein surfaces cannot always be predicted easily, which limits access to more complicated 3D protein structures.

Endogenous small molecules are known to bind to various proteins by protein–ligand interactions, which has also been exploited to prepare structurally defined PNs. The most prevalent examples for natural ligand-guided PNs are heme proteins, (strept)avidins and lectins that bind heme, biotin, and carbohydrates (mannose, glucose, and galactose), respectively. Most of these interactions are highly specific and exhibit reasonable binding affinities from the micromolar (μM) to the femtomolar (fM) regime as listed in Table 2. These unique binding features have been exploited in a range of applications and most commonly in affinity purification.^36^,^37^

**Table 2:** Summary of SLs and stability or binding constant with the corresponding recognition motif

Interaction motif	SL	Stability/binding constant
Charge-directed assembly	Gly-Val-Gly-Lys-Pro	Complete disassembly [NaCl] > 100 mM
	Newkome-type dendrimer	Photocleavable
	Phthalocyanine	Not determined
	Cationic diblock copolymers	Disassembly at *T* < 40 °C
	Cationic CdTe quantum dots	Some disassembly at [NaCl] > 250 mM
	G5-PAMAM dendrimers	Disassembly at [NaCl] > 400 mM; pH < 2; pH > 12
	Cationic cross-linked micelles	Not determined
Heme–hemeprotein	Heme	*K*_a_ ∼ 10^12–14^ M^–1^
Lectin–carbohydrate	Galactose	*K*_a_ ∼ 10^3^ M^–1^
	Tetra-d-galactose	*K*_a_ ∼ 10^9^ M^–1^
	α-d-Mannose	*K*_a_ ∼ 10^3^–10^6^ M^–1^
	*N*-Acetyl-α-d-galactosamine	*K*_a_ ∼ 10^4^ M^–1^
	α-d-Galactopyranoside	*K*_a_ ∼ 10^4^ M^–1^
(Strept)avidin–biotin	Biotin–hydrazone-linker	Cleavage at pH < 7
	Iminobiotin	pH > 7: *K*_a_ ∼ 10^11^ M^–1^; pH < 7 : 10^3^ M^–1^
Boronic acid	Salicylhydroxamic acid	pH > 7: *K*_a_ ∼ 10^6^ M^–1^; pH < 7 : 10^3^ M^–1^
Phe-Gly-Gly	CB[8]	*K*_ter_ ∼ 10^11^ M^–2^
Naphthalene–methyl viologen	CB[8]	*K*_1_ ∼ 10^5^ M^–1^; *K*_2_ ∼ 10^6^ M^–1^
Arg128 (lysozyme)	*p*-Sulfonato-calix[4]-arene	*K*_a_ ∼ 10^6^ M^–1^
*i* + *i*4 histidine motifs	Zn^II^	Disassembly pH < 5; in presence of EDTA
Spiropyran	Mg^II^	Disassembly with mechanical force; in presence of EDTA
5′-GCTACACG-3′ (8-mer)	3′-CGATGTGC-5′	*K*_a_ ∼ 0.1 × 10^6^ M^–1^
5′-AGCTACACGATA-3′ (12-mer)	3′-TCGATGTGCTAT-5′	*K*_a_ ∼ 9 × 10^9^ M^–1^
5′-AAAAAAAAAAAA-3′ (12-mer)	3-TTTTTTTTTTTT-5′	*K*_a_ ∼ 0.1 × 10^6^ M^–1^

Lectins are carbohydrate binding proteins, which are involved in a number of cellular processes including glycoprotein synthesis, modulating inflammatory responses, and cell recognition.^38^ Plant lectins such as concanavalin A and lectin A are homotetrameric proteins with four binding sites for mannose, glucose, or galactose.^39^–^41^ Concanavalin A, a tetrameric protein with D2 symmetry binds to terminal α-d-mannosyl and α-d-glucosyl groups and was first used by Freeman *et al.* in combination with bismannopyranoside (chemical structure shown in red inset, Fig. 2c) as SL to form predesigned, diamond-like protein lattices.^42^ Interestingly, the choice of the lectin unit has a strong influence on the resultant nanoarchitecture. For instance, the tetrahedral concanavalin A (Fig. 2c) formed an interpenetrating protein crystalline framework when assembled with a bifunctional linker comprising of a sugar and rhodamine B.^41^ However, in the case of the homotetrameric soybean agglutinin possessing a *D*_2_ symmetry with slightly out of plane binding pockets (Fig. 2d), a microtubule-like structure was obtained.^43^ This feature was attributed to the difference in protein geometries of concanavalin A and soybean agglutinin.^43^ Besides the plant lectins described above, human Galectin-1, a lectin from animal source, was found to form self-assembled microribbons using this strategy.^44^

Avidin and streptavidin are homotetrameric proteins containing eight β-strands in each subunit, resulting in an antiparallel β-barrel shaped structure (Fig. 2e). The proteins are proposed to inhibit bacteria growth and bind to vitamin B_7_, biotin, in a non-covalent manner with one of the strongest binding affinities known (*K*_a_ ∼ 10^15^ M^–1^). The biotin–(strept)avidin interaction has been exploited for various biological applications including purification^45^ and cancer pretargeting^46^ as well as a supramolecular “glue” for building up various nanostructures^47^ and forming protein networks.^48^ Supramolecular assembly to form linear avidin polymers has been achieved using bis-biotinyl linkers (Fig. 6e) and the spacer length affected the stability of the resultant polymer as discussed in Section 2.2.2.^49^ However, such avidin polymerizations are often uncontrolled,^50^ and the resultant materials have no specific functions. More recently, synthetic efforts have allowed the creation of spatially defined supramolecular protein nanostructures with avidin through tailored linker design, which are discussed in Section 2.2. Lectins and (strept)avidin PBs do not possess any bioactivity and additional functionality has to be incorporated either by surface modifications or by co-assembly with other functional entities such as protein enzymes or synthetic molecules such as dendrons as described in Section 2.3.^51^

**Fig. 3:**
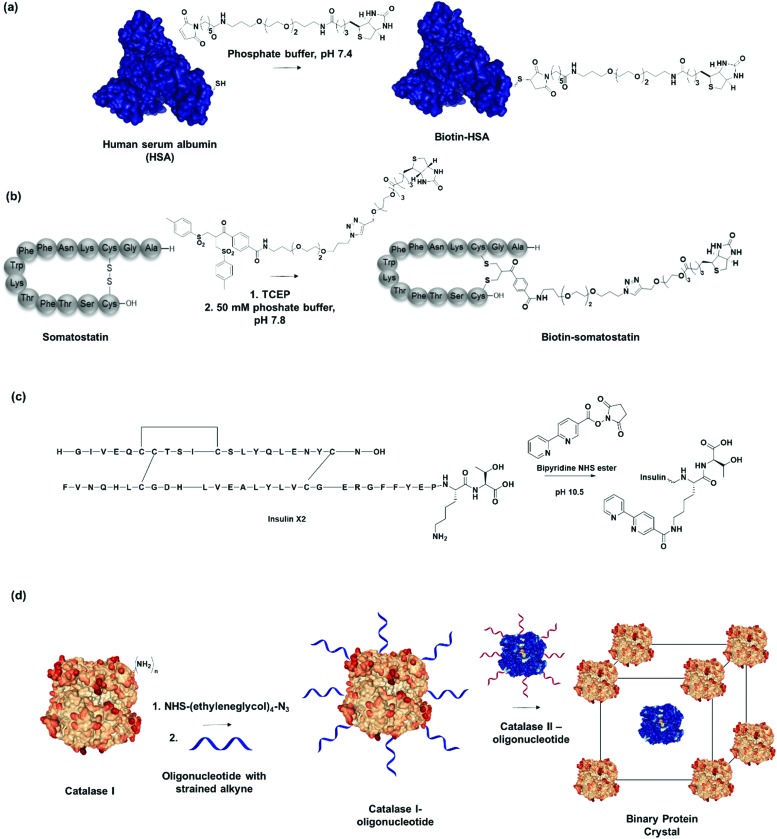
(a) Human serum albumin (HSA) modified with recognition motifs at single unpaired cysteine (Cys-34). Adapted with permission from ref. 20. Copyright 2013 American Chemical Society. (b) Biotin recognition motif incorporated through disulfide modification on the hormone peptide, somatostatin. Adapted with permission from ref. 62. Copyright 2018 Wiley-VCH Verlag GmbH & Co. KGaA, Weinheim. (c) Insulin variant (Insulin X2) modified with bipyridine recognition motif at LysB29N^ε^. (d) Two different catalase PBs modified with complementary oligonucleotide sequences assembles into a binary crystalline structure. Adapted with permission from ref. 74. Copyright 2015 National Academy of Science.

Clearly, protein interfaces and pre-existing protein recognition units are valuable to induce non-covalent interactions between PB to obtain PNs with well-defined structures. However, the repertoire of native PBs is limited by the structures Nature offers. In order to further expand the plethora of PBs, alternative approaches are required. In this regard, the incorporation of a recognition unit, usually an endogenous ligand such as biotin, deoxyribonucleic acid, or peptide; or exogenous molecules that undergo host–guest complex formation, is the method of choice to control the assembly process. In this case, site-directed conjugation of a recognition motif to the protein of interest is required to impart the chemical information for assembly, which is discussed in the next section.

#### 2.1.2 Chemically modified PBs

Many proteins provide amino acid side chains that can be employed for chemical functionalization. However, in order to construct defined PNs, chemo- and regio-selectivity of the bioconjugation reactions represent a prerequisite as well as mild reaction conditions. Currently, reengineering protein surface recognition and even imparting novel functions by applying site-selective chemical methodologies under “protein-friendly” conditions represent established methods to form precise bioconjugates.^17^,^52^,^53^ The relatively rare amino acid cysteine is most often exploited due to the selective reactivity of its thiol group towards maleimides under controlled pH.^54^ The abundant blood plasma proteins human or bovine serum albumins (HSA, BSA) provide Cys34 modified through maleimide-thiol Michael reaction (Fig. 3a). For example, a single biotin ligand has been attached to HSA at neutral pH to avoid side reactions with the primary amino groups of lysine residues.^20^ Nevertheless, very few native proteins contain a single or even two unpaired and accessible cysteine residues at their surfaces.^55^,^56^ Most cysteines in proteins are present in the reduced form as disulfides. A broad range of proteins^57^–^59^ offers accessible disulfide bonds that control their stability and biological activity^60^ and that can be modified by disulfide rebridging reagents, such as bissulfone (Fig. 3b) or allyl sulfone reagents.^61^,^62^ Using this functionalization approach, the cyclic peptide hormones somatostatin and insulin as well as protein enzymes like lysozyme consisting of one, three and four disulfide bonds, respectively, have been equipped with a single recognition motif such as biotin or a boronic acid, which interacts with carbohydrates.^61^,^63^,^64^

Thulstrup *et al.* attached a single bipyridine into insulin through specific modification of the lysine residue in chain B (LysB29) (Fig. 3c).^65^ Insulin has one lysine, LysB29, with a p*K*_a_ of 11.2 for LysB29N^ε^, whereas the N^α^ on the A and B chain provide p*K*_a_s of 8.4 and 7.1, respectively. By exploiting the difference in p*K*_a_, selective acylation at LysB29 was achieved in basic conditions at pH > 10.^65^ Conversely, at physiological pH, the Lys side chain is the least reactive amine but bioconjugation strategies have been reported that also allow N-terminal modification.^66^,^67^ Tyrosine and tryptophan conjugations have been applied to a broader spectrum of proteins but these residues are normally less accessible as they are often buried in the hydrophobic interior of proteins.^68^,^69^ Thus, a single site can be easily introduced to place an external recognition motif onto the protein of interest. For instance, bifunctional linkers with click functional groups were used to prepare protein–DNA conjugates through binding onto tyrosine residues.^70^ In principle, the site-selective chemical modification strategies mentioned are also applied to introduce SLs to proteins of interest.^61^ Dual functionalization strategies have also been developed in a site-directed fashion, for example by capitalizing on reactivity variations of different cysteine residues^71^,^72^ or in combination with N-terminal protein modification.^73^ By the careful selection of the PB and the chemical modification method, it will be possible to attach two different recognition motif in a defined spatial orientation. Consequently, it would allow the generation of nanostructures consisting of two different PBs in a controlled, stepwise manner.

Previously, site-selective incorporation of the recognition motif to induce directionality was considered a prerequisite for nanostructure formation. However, Mirkin, *et al.* demonstrated that statistically modified proteins can also be employed as PBs to engineer multi-enzyme crystals due to their distinct pattern of surface-accessible amine groups and the consistency of surface morphology on a rigid protein core.^74^ In this manner, they modified two tetrameric heme catalase enzymes with oligonucleotides over two step chemical reactions, first by adding a tetraethylene glycol linker functionalized with *N*-hydroxysuccinimide (NHS) ester and azide on both ends to connect to the amines on the catalase, followed by a cycloaddition with an oligonucleotide functionalized with dibenzocyclooctyne at the 5′-end (Fig. 3d). Protein–DNA PBs with functional densities of 30–50 pmol cm^–2^ were obtained in this fashion.^74^ By preparing two DNA-modified PBs with complementary oligonucleotide sequences, they demonstrated that multienzyme protein crystals, which retain catalytic function of the individual PBs, were prepared in a straightforward fashion.^74^

Even though a variety of protein bioconjugation techniques exist nowadays, these methods have surprisingly not been used as first choice to reengineer PBs for ultimate assembly of PNs. One plausible explanation could be the widespread application of thiol-maleimide reactions and the ease of engineering cysteine mutants for the desired PB. Nonetheless, chemical toolboxes offer versatile incorporation of non-endogenous ligands into proteins, and with the expanding repertoire of these tools, this strategy will gain even stronger footholds in the future.

#### 2.1.3 Genetically modified PBs

**Fig. 4:**
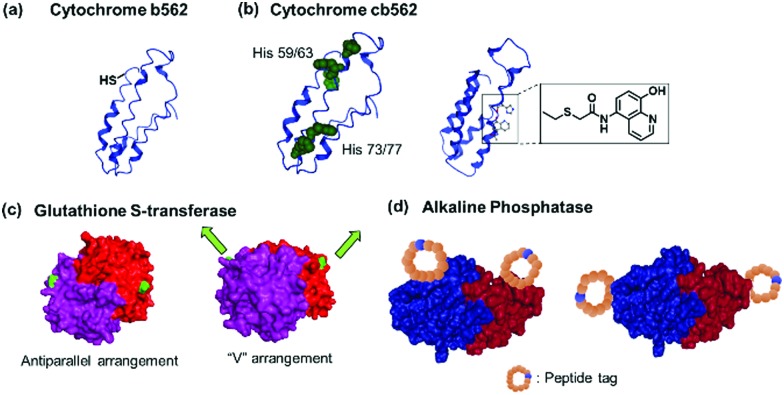
Genetically modified PBs. (a) Cytochrome *b*_562_ 63Cys mutant with unpaired thiol. (b) Cytochrome *cb*_562_ variants with *i*/*i* + 4 His (left) or quinolate (right) mutations. Adapted with permission from ref. 79 and 80. Copyright 2007, 2010 American Chemical Society. (c) Glutathione-*S*-transferase with mutations in antiparallel or “V” orientation. Adapted with permission from ref. 29 and 84. Copyright 2017, 2012 Royal Society of Chemistry. (d) Alkaline phosphatase with mutations incorporating reactive peptide tags along the short or long axis of the protein. Adapted with permission from ref. 85 and 86. Copyright 2011 Royal Society of Chemistry. Protein images (PDB: ; 1EW9) were adapted from the NGL viewer.^240^

Genetic engineering is widely applied to express PBs with a single mutation *e.g.* cysteine. For instance, linear polymers of hemeproteins cannot be formed as this protein only provides one binding pocket. To circumvent this issue, a single point cysteine genetic mutation was introduced onto the hemeprotein and subsequently, a heme group equipped with a site-selective cysteine modification was incorporated into cytochrome *b*_562_ (Fig. 4a).^27^ This incorporation allowed the formation of the first supramolecular linear hemeprotein polymer, as reported by Hayashi, *et al.*^27^ Other hemeprotein PBs, such as myoglobin, have been prepared using a similar approach.^75^ Protein homodimers have been formed through dynamic covalent disulfide bonds *via* recombinant technologies to generate AA type PB, *i.e.* PB consisting of two similar units, for linear polymerization.^76^ In theory, this method could also be applicable to prepare AB-type PB comprising of hetero-heme protein dimers that could be further assembled to generate supramolecular protein polymers with precise alternating arrangement.

Peptide sequences that interact with external molecular triggers such as host–guest interactions or through metal coordination were introduced *via* genetic engineering. The short peptide tag, Phe-Gly-Gly, is known to form an inclusion complex with cucurbit[8]uril (CB[8]) in a 2 : 1 ratio.^77^,^78^ This tag has been introduced into fluorescent proteins such as cyan fluorescent protein (CFP) or yellow fluorescent protein (YFP) *via* the N- or C-termini.^77^,^78^ Similarly, insertion of sets of histidine residues *via* genetic engineering on the proteins of interest, such as within cytochrome C's α-helixes (Fig. 4a), has proven to be particularly suitable for assemblies formed through metal coordination.^6^,^79^ Tezcan and coworkers genetically modified a cytochrome *cb*_562_ variant containing histidine residues capable of coordinating onto a metal ion such as Zn^2+^.^79^ Here, two sets of His-dimers were inserted in positions 59/63 and 73/77 of the helix 3 of cytochrome *cb*_562_.^79^ Hybrid coordination motifs have also been achieved using a combination of a natural histidine and non-natural quinolate amino acid (Fig. 4b) as chelating ligands.^80^ Mutations of amino acids in the protein sequence were also applied to remove unwanted interactions and guide the self-assembly process. For example, amino acid groups located at *C*_3_ symmetry positions on a ferritin cage were substituted by histidine (Thr122His).^81^ To prevent other types of coordination rather than by the histidine residues, the mutations Cys90Gln, Cys102Ala, Cys130Ala, and Lys86Gln were also performed to subsequently generate a highly ordered crystalline metal–organic protein framework.^81^ Nevertheless, such strategy requires a profound knowledge of the 3D structures of the proteins and of the active site in order to devise a reasonable design. Additionally, the process from design to executing the mutations could be tedious.

Besides the introduction of a single recognition site, genetic engineering also allowed for the placement of multiple copies of the recognition motifs to direct the spatial orientation of the protein nanoassembly. Tobacco mosaic virus coat protein mutants with two cysteine or four histidine residues at the lateral surfaces were prepared and by controlling the thermodynamics and kinetics of the respective PBs, the crystal structures of the PN were tailored. The cysteine mutant formed triclinic crystals at 4 °C over a month, while the histidine mutant rapidly assembled in the presence of Zn^2+^ to form hexagonal close-packed crystals.^82^ Specific cysteine mutations were performed on chaperonin GroEL, a protein that mediates protein folding in cells, to generate GroELCys with 14 Cys residues spatially distributed on the top and bottom of the cylindrical shape protein.^83^ Subsequent functionalization with maleimide recognition motif allowed the directionality of protein polymerization to be controlled.^83^ Glutathione-*S*-transferase (GST) from *Schistosoma japonicum* forms homodimers, and genetic fusion of either the hexahistidine or Phe-Gly-Gly tag on the N-termini^84^ yielded an antiparallel arrangement of the recognition motif or the fusion tag was arranged in a “V” shape (Fig. 4c),^29^ which affected the final morphology of the protein nanostructures.

Kamiya *et al.* dictates the directionality of the self-assembly through control of the placement of the modification sites on a functional alkaline phosphatase by recombinant engineering.^85^,^86^ Microbial transglutaminase-catalysed acyl-transfer reaction is known to occur between the side chains of glutamine and lysine amino groups. Thus, the predefined positioning of a transglutaminase-reactive peptide tag at the N- or/and C-terminal allowed biotinylation in a specific orientation on the alkaline phosphatase.^85^,^86^ However, the chain growth was terminated presumably due to steric crowding since both the N- and C-termini were facing in the same directions. Further optimization of the placement of a microbial transglutaminase tag along the longer axis of the alkaline phosphatase circumvented this issue and allowed intermolecular polymerization (Fig. 4d).^86^ To confer additional self-assembling handles on streptavidin, a twigged streptavidin polymer was engineered by Tanaka and co-workers as a scaffold for hetero-protein assembly.^30^ A sortase A recognition site and a horseradish peroxidase recognition site were genetically incorporated into the N- and C-termini of streptavidin, respectively, that allowed the immobilization of two different proteins *via* biotin–streptavidin interaction and sortase A-mediated ligation.^30^

Genetic engineering provides many opportunities concerning the introduction of functionalities at distinct sites of PBs that chemical modification alone cannot achieve and *vice versa*. However, recombinant technologies also have some drawbacks in terms of laborious processes, loss of protein activity due to structure changes based on the introduced mutation and lack of possibility to include PBs that have been chemically post-modified to expand and customize the functional profile of the protein nanostructures. Thus, in the long term, the combination of both chemical and biotechnological toolboxes will provide entirely new supramolecular protein nanostructures with customized geometries and features.

### 2.2. Design of the supramolecular linkers (SLs)

The supramolecular linker (SL) often functions as a “glue”, which interacts specifically with the recognition motif on the PBs to direct the formation of the desired PN. Thus, the assembly and the resultant architectures and stabilities could be strongly influenced by the design of the SLs as well as by the choice of the suitable recognition motif. These two factors are often co-related and are featured together in this section. In some cases, the SLs attached directly to the PBs to induce PN formation, but in other instances, conjugation to PBs was required and some examples will be given. A summary of selected SLs and complex stabilities or binding constant with the corresponding recognition motif, as well as chemical structures of relevant SLs are given in Fig. 6 and Table 2. In the last part of Section 2.2, methods for quantification of the interactions are highlighted.

#### 2.2.1 SLs based on electrostatic interactions

As discussed in Section 2.1.1, binary crystals were obtained from oppositely charged PBs but cannot be applied to proteins with similar charges.^34^ To expand further on this strategy,^34^ engineered PBs, synthetic macromolecules or nanoparticles have been used as SL to form heteroassemblies. In order to induce interfacial electrostatic interactions, these non-natural SLs should possess compatible sizes and shapes (generally globular) to the PBs.^35^,^87^–^92^ This strategy offers flexibility to control assembly and disassembly due to the sensitivity of charged interactions towards pH value and ionic strength.^87^,^91^,^92^ In this approach, the SLs could be applied without the need for covalent conjugation to the PBs. Additionally, due to the nature of the charge interactions between SL–PB, assembly and disassembly of PNs could often be controlled through variation of the ionic strength or pH of the buffer used (Table 2).

**Fig. 5:**
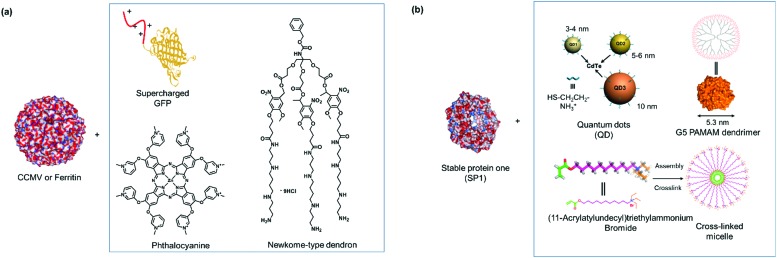
(a) SLs that are applied with negatively charged CCMV or ferritin. Adapted with permission from ref. 87, 88 and 90. Copyright 2016, 2018 American Chemical Society, 2010 Nature Publishing Group. (b) SLs used for forming PNs with stable protein one (SP1). Adapted with permission from ref. 35, 91 and 92. Copyright 2014, 2015, 2016 American Chemical Society.

For instance, new polycationic surface areas were introduced by expressing positively charged peptide sequences into negatively charged proteins or by using positively charged synthetic macromolecules. For instance, the sequence Gly-Val-Gly-Lys-Pro was fused to the sequence coding for a green fluorescent protein (GFP) to form a supercharged cationic polypeptide (Fig. 5a) that formed a new polycationic surface patch at distinct location at the GFP surface.^87^ Alternatively, the assembly of PNs with highly branched polycationic Newkome-type dendrons was reported.^88^ Dendrons are branches of dendrimers, which are monodisperse globular synthetic macromolecules that resemble proteins in certain structural characteristics such as their sizes, globular architecture and the availability of many polar groups at their surface and unipolar groups within the interior.^93^ Positively charged dendrons with photocleavable *o*-nitrobenzyl have been introduced as SLs that bind to the negatively charged surface patches of CCMV.^88^ The cleavage of the SL is an irreversible chemical process and reassembly is not possible. Large ordered PN architectures consisting of (apo)ferritin cages or CCMV were formed that revealed unique characteristics such as light-induced disassembly of PNs (Fig. 5a).^88^ Interestingly, the responsive behavior of the resultant nanostructure was fine-tuned by structural alterations of the cationic inducer. For instance, replacing the dendron with a cationic diblock copolymer introduced a thermo-switch so that the protein nanostructure became responsive to temperature changes.^89^ Torres *et al.* prepared a tetracationic complex, formed from an octacationic zinc phthalocyanine (Fig. 5a) and a tetraanionic pyrene, as a SL. The SL also produced additional function as a photosensitizers for the formation of singlet oxygen, a crucial process for photodynamic therapy.^94^

PNs consisting of “heteroassemblies” of PBs and macromolecules such as dendrimers or nanoparticles like quantum dots possessing similar sizes and globular shapes as the PBs have been achieved.^91^,^92^ Liu *et al.* have successfully employed cationic, hard nanoparticles such as quantum dots and soft particles such as dendrimers and micelles (Fig. 5b) to derive a series of functional self-assembled heteroprotein complexes, showing the immense potential of this strategy for both biomedical and biotechnological applications.^35^,^91^,^92^ In their earliest report, positively charged, globular quantum dots of various sizes were synthesized, and the impact of the quantum dot sizes on PNs was investigated.^91^ Further to this development, an electro-positively charged macromolecule, the fifth generation polyamidoamine (G5 PAMAM) dendrimer was used in place of quantum dots to control the self-assembly process of cricoid SP1 and protein nanorods were formed.^92^ Dendrimer component offers the advantage that assembly efficiency and morphology of the PNs could be adjusted precisely by the dendrimer generation (size) and scaffolds. Moreover, dendrimers also provided additional level of functionality and in the above example, G5 PAMAM was functionalized with manganese porphyrin to confer superoxide dismutase activity. In this case, the stability of the SL–PB interaction is affected by ionic strength and pH of the buffer. For instance at pH < 2 and pH > 12 or at higher salt concentrations (400 mM NaCl), the interactions are much weaker and dissociation could be observed. Notably, temperature does not have much effect on the stability of the interactions^92^ In order to improve on the ease of preparation and customization of the SL, the same group explored the feasibility of using core cross-linked micelles with cationic surfaces as inducers for self-assembly.^35^ They demonstrated that the cross-linked micelles were functionalized in a convenient manner with chromophores to introduce additional functionality to the system.^35^

Overall, SLs that bind *via* electrostatic interactions offer a convenient and versatile strategy as additional functional features can be introduced to the PN by molecular design and association/dissociation is dependent on and thus, tunable by ionic strengths. However, challenges remain in terms of control of the charge distribution on the complicated 3D protein surface, which can be difficult to predict and to manipulate and if not optimal, it compromises the spatial organization of the PN.

#### 2.2.2 SLs based on protein–ligand interactions

In earlier examples, a linker consisting of a protein-binding ligand induced protein polymerization yielding well-defined PNs.^42^,^43^ However, the morphology of the PNs was determined by the PB, and imparting structural variations was challenging. This has stimulated several attempts to design supramolecular linkers providing directionality in their interactions to control the formation of more complex protein architectures.

**Fig. 6:**
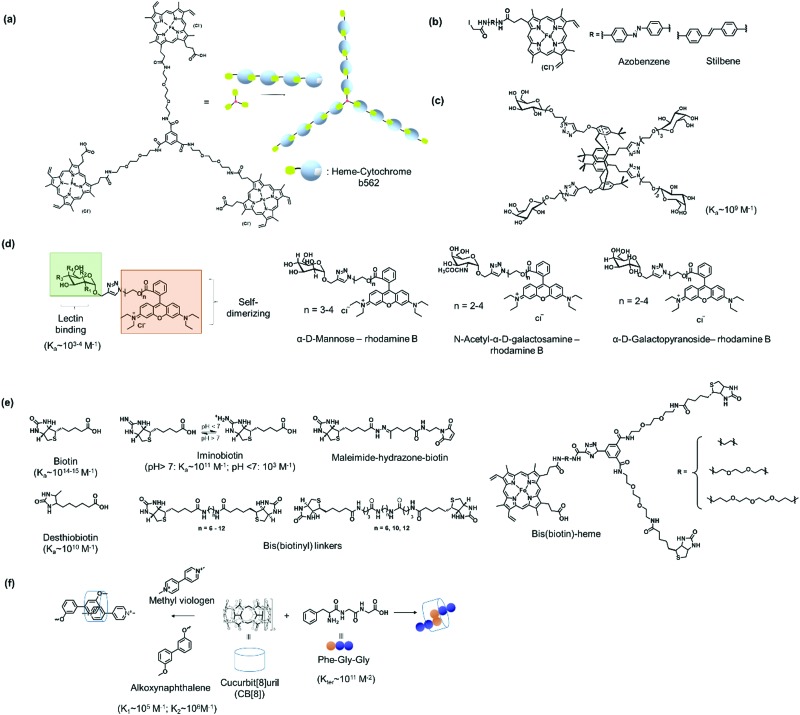
Design of selected SLs described and reference values for binding constants. (a) Tris(heme) SL based on a C3 phenyl core. Adapted with permission from ref. 96. Copyright 2009 Wiley-VCH Verlag GmbH & Co. KGaA, Weinheim. (b) Thermoresponsive heme SL with rigid stilbene or azobenzene tethers. (c) A tetra-galactosylated glycocluster with increased binding affinity to lectin A. (d) Bifunctional SLs with rhodamine B and different sugar moieties. (e) SL based on biotin and biotin derivatives. (f) CB[8] SLs with Phe-Gly-Gly or synthetic chemical ligands for ternary complex formation.

The high affinity of heme for apoglobin proteins (equilibrium dissociation constant: 10^–12^–10^–15^ M^95^) has been exploited for formation of linear protein polymers.^27^,^75^ To further exploit this protein–ligand interaction, Hayashi *et al.* have adopted a C3-phenyl core to design a heme triad linker to introduce a branching point (Fig. 6a), resulting in the formation of two dimensional networks with cytochrome *b*_562_.^96^ Transient thermal stimuli consisting of rigid and hydrophobic tethering group such as azobenzene or stilbene was also incorporated into the artificial heme SL so that a switch between different PNs was achieved (Fig. 6b).^97^ On the other hand, SLs comprising of phenyl and octyl moieties as tethering groups dissociate upon heating. It was proposed that the azobenzene/stillebene moieties offer stabilization of the metastable micellar structure even after cooling, through π–π and/or C–H–π interactions with the heme PB.^97^

Multivalent linkers based on a rigid core were also functionalized to control the topology as well as to enhance the binding affinity between the PB and the SL. For instance, the monovalent galactose ligand typically exhibits binding affinity to lectin A in the submillimolar range.^98^ Based on multivalent “glycoside cluster effect”, a tetra-galactosylated glycocluster was synthesized (Fig. 6c), in which the galactose ligand reveals significantly enhanced binding (nanomolar) to lectin A.^99^,^100^ Jiang *et al.* have further devised a lectin-carbohydrate driven PN formation strategy *via* a combination of dual supramolecular interactions to achieve variations in PN morphology and to incorporate stimuli-responsiveness.^40^,^41^,^43^ Several SLs have been prepared for the formation of a variety of PNs. These linkers typically consist of a rhodamine B group, which forms π–π interactions with another rhodamine B molecule, an oligo(ethylene oxide) spacer to control the distance between interacting PBs, and a sugar unit such as α-d-mannose of *N*-acetyl-α-d-galactosamine or α-d-galactopyranoside (Fig. 6d) for interactions with the corresponding lectins such as concanavalin A,^41^ lectin A,^40^ or soybean agglutinin.^43^ The inclusion of an oligo(ethylene oxide) spacer also allows varying the SL length, which was found to strongly influence the assembly process.^40^,^41^ For example, a SL consisting of a monosaccharide (*e.g.* α-d-mannose) and a rhodamine B group was used to crosslink concanavalin A *via* both lectin-carbohydrate and π–π interactions resulting in the formation a protein crystalline framework.^41^ The incorporation of rhodamine B in the SL design allows reversible association and dissociation of the PNs through competitive host–guest interactions using β-cyclodextrin capable to interact with rhodamine B.^43^ In this case, dissociation is achieved and re-association can be induced by using 1-adamantane hydrochloride, a competitive binding ligand.^43^

Of all the systems exploiting protein–ligand interactions, the (strept)avidin–biotin system offers the greatest flexibility in the library of ligands (Fig. 6e) that are available to confer high binding strengths together with stimuli-responsiveness. Binding affinities of biotin to strept(avidin) are one of the strongest protein–ligand interactions known and could be varied through chemical variations. For instance, desthiobiotin (*K*_a_ ∼ 10^10^ M^–1^)^101^ and pH sensitive iminobiotin (pH > 7, *K*_a_ ∼ 10^11^ M^–1^; pH < 7, *K*_a_ ∼ 10^3^ M^–1^)^102^ reveal altered binding characteristics for (strept)avidin when compared to biotin. The lower binding affinities of these commercially available biotin analogues offer the possibility to control association and dissociation with PB by variation of reaction conditions.^20^ In particular, iminobiotin provides dynamic and reversible binding to (strept)avidin as the imine is protonated at acidic pH, which strongly reduces binding to (strept)avidin.^103^ Biotin analogues consisting of the redox sensitive S–S or the pH sensitive hydrazone bond are commercially available and offer straightforward customizations of SL for the formation of stimuli-responsive PNs *e.g.* to react to the more acidic microenvironments of diseased cells.^62^ Besides responsiveness, SL design with dynamic covalent S–S and hydrazone linkage offers at the same time, the stability of covalent bonds.^104^ Moreover, synthetic heterobifunctional biotin conjugation reagents, such as pH cleavable maleimide-biotin SLs, have also been reported in the literature.^62^ Here, the maleimide-biotin SLs are attached to the protein on interest by conjugation through its unpaired cysteine.^62^

Bis(biotinyl) SLs (Fig. 6e) have been prepared with different chain lengths with up to 25 bonds between the carbonyl groups on the biotin.^49^ It was found that the number of bonds affected the stability of the resultant linear polymers.^49^ When the number of bonds were higher than 12, the SL could crosslink two avidin PBs and yielded linear PNs.^49^ PNs with 12–13 bonds were less stable and disassembled while PNs formed with longer chain reagents were more stable in the presence of competing biotin ligands.^49^ However, when a critical length of 23 bonds was reached, polymerization did not occur and the SL bound to two ligand sites on a single avidin PB.

Bifunctional SL consisting bis(biotin) and a heme ligand, for instance, have been designed to control spatial arrangement of the individual PBs (Fig. 6e).^105^ The bis(biotin) moiety bound specifically to the two adjacent biotin binding sites of streptavidin and thus preorganized the assembly in a linear fashion.^105^ Consequently, it was shown that the bis(biotin)-heme SL could be used to crosslink the proteins streptavidin (B) and apomyoglobin (AA) to form a linear AAB type supramolecular PN with precise alternating protein arrangement.^105^

#### 2.2.3 SLs based on synthetic host–guest interactions

Besides natural binding ligands, synthetic interactions have also been introduced into PBs to control assembly, as exemplified by host–guest interactions such as that of cucurbit[8]uril (CB[8]), a macrocyclic molecule made of glycoluril monomers linked by methylene, with peptides or aromatic ligands^77^ (Fig. 6f) and β-cyclodextrin with lithocholic acid.^28^ One could envisage that such exogenous ligands be potentially applied to self-assemble in complex cellular environments due to their bioorthogonality and high complex stability, while reversibility could be achieved *via* interplay of binding strengths of SL, PBs and competitive binding ligands.

Protein homo- and hetero-dimerization has been reported using synthetic host–guest interaction.^28^,^77^,^78^,^106^,^107^ The “ligands” in these cases were either obtained by genetic engineering, as in the case of the peptide such as Phe-Gly-Gly (CB[8] : Phe-Gly-Gly = 1 : 2, *K*_ter_ = 1.5 × 10^11^ M^–2^),^77^,^108^ usually at the N-terminus or in the case of synthetic entities such as lithocholic acid, introduced site-selectively on a single cysteine mutant of the PB, forming a host–guest complexes with β-cyclodextrin.^109^ The addition of ligands such as methyl viologen (Fig. 6f) that also interacts strongly with CB[8] (*K*_a_ ∼ 10^6^ M^–1^), results in competitive binding and PN dissociation.^77^ Ternary complexation can also be programmed by the introduction of recognition motifs that interact with CB[8] in a 1 : 1 : 1 manner, for example, with PBs consisting of an electron deficient supramolecular guest molecules such as methyl viologen and a complementary electron rich guest such as alkoxynaphthalene (Fig. 6f),^106^ where the CB[8]:ligand binding (methyl violgen and alkoxynaphthalene, respectively) are in the range of *K*_1_ ∼ 10^5^ M^–1^ and *K*_2_ ∼ 10^6^ M^–1^.^110^ A combination of chemical and recombinant techniques adopting the cyclodextrin or CB[8] host–guest interaction offers a powerful platform to tailor a variety of multiprotein assemblies with different structural features.

Calix[*n*]arenes are symmetrical macrocycles possessing four phenolic arms and are typically cone-shaped with defined upper- and lower-rim regions. Their secondary and ternary interactions have been extensively studied and are relevant for various supramolecular assembly and crystal engineering processes.^111^ In a biological context, the anionic, water-soluble derivatives such as *p*-sulfonato-calix[4]-arene and *p*-phosphonatocalix-[6]arene are emerging as candidates to drive protein assembly by electrostatic interactions due to the possibility for molecular recognition through encapsulation of the cationic side chains of lysine and arginine.^112^ For instance, Crowley *et al.* reported the complexation of the hen's egg white protein, lysozyme, with *p*-sulfonato-calix[4]-arene to form a linear assembly of protein tetramers.^113^ The macrocyclic *p*-sulfonato-calix[4]-arene serves as SL between the protein units *via* twofold interactions: (1) encapsulation of the C-terminal Arg128 owing to its steric accessibility and (2) forming a protein-bound complex of *p*-sulfonato-calix[4]-arene, Mg^2+^, and a polyethylene glycol, which is present in the reservoir solution for crystallization. The same group also applied the larger macrocyclic *p*-phosphonatocalix-[6]arene to form a symmetric C2 protein dimer of cytochrome *c*.^114^ Subsequently, a sulfonate-calix[8]arene SL, which offered greater conformation flexibility, mediated the formation of cytochrome *c* tetramers in solution. Auto-regulation of assembly and disassembly could be achieved through the control of the SL concentration, without the need to use competitive ligand for inhibition.^115^ Liu *et al.* used a sulfato-β-cyclodextrin, which has a higher negative charge density, to form spherical nanoparticles (100–160 nm) with the cationic DNA-binding protein protamine.^116^ The particle formation is determined to arise from the charge surface rather than any single amino acid residues alone. The sulfato-β-cyclodextrin/protamine nanoparticles could be degraded by trypsin enzyme for controlled release of cargoes.^116^

#### 2.2.4 SLs based on metal–ligand interactions

Metal ions such as Ca^2+^, Fe^*n*+^, Co^*n*+^, Ni^*n*+^, Cu^*n*+^, and Zn^*n*+^ (*n* = 1, 2 or 3) play crucial roles in biological systems.^117^–^119^ They can bind to proteins by metal–ligand coordination with certain amino acids usually bearing a lone pair such as histidine (His), cysteine (Cys), and aspartic acid (Asp) to form coordination bonds.^6^,^79^,^120^ It was proposed that the higher bond strength of metal–ligand coordination could overcome weaker interactions, such as hydrogen bonds, electrostatic, or non-covalent bonds, resulting in more selective and high binding affinities, which could impart greater control of the assembly process and higher stability of the formed PNs.^6^,^121^ The complex geometry, bond strengths, and functions are affected by the type of ancillary ligands, the oxidation state of the metal ions, and their respective coordination numbers.^5^ Thus, the directionality of the coordination bonds, the labilities and the properties of the resultant PNs can be fine-tuned. In the past decade, several approaches have emerged, where metal ions were used as supramolecular “glue” in combination with a suitable coordinating ligand to derive unique PNs.^84^,^122^,^123^ The detailed description of the concept of metal-directed protein assembly has been covered in a review,^6^ and only selected examples are further discussed to highlight the main PN design strategies based on coordination chemistry.

**Fig. 7:**
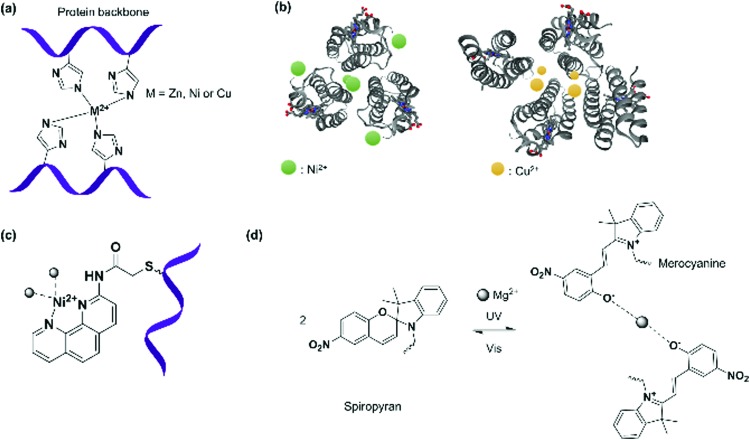
(a) Bis(histidine) complexation to metal ions. (b) Antiparallel *C*_2_-symmetric dimers of the type 2Cu^2+^:2 cytochrome *cb*_562_ or *C*_3_-symmetric trimers of the type 2Ni^2+^:3 cytochrome *cb*_562_ formed due to influence of the respective metal ions on the coordination geometry. Adapted with permission from ref. 124. Copyright 2009 American Chemical Society. Protein images (PDB: ; 3DE8, ; 3DE9) were adapted from the NGL viewer.^240^ (c) Complexation of PB modified with 1,10-phenanthroline to Ni^2+^ ion. (d) Light driven coordination of Mg^2+^ with spiropyran/merocyanine ligands.

Four cytochrome *cb*_562_ variant consisting of two sets of His dimers inserted at the *i* and *i* + 4 positions (Fig. 4b and 7a) assembled into two interlaced V-shaped dimers in an antiparallel fashion to one another in the presence of Zn^2+^ ions.^79^ Dissociation of the nanostructure was induced through a change in pH (≤5) or the addition of strongly chelating ligands such as ethylenediaminetetraacetic acid (EDTA).^79^ Variation of the Zn^2+^ concentration in solution yielded monomers and dimers, whereas the formation of tetramers or even higher order polymers occurred at high concentrations.^79^ By replacing the Zn^2+^ ions with Cu^2+^ (square geometry) or Ni^2+^ (octahedral geometry),^6^,^124^ PNs comprising of antiparallel *C*_2_-symmetric dimers of the type 2Cu^2+^:2 cytochrome *cb*_562_ or *C*_3_-symmetric trimers of the type 2Ni^2+^:3 cytochrome *cb*_562_ were formed, respectively, presumably due to the coordination geometry imposed by the metal ions (Fig. 7b). By variation of the His59 and Cys96 mutation on the cytochrome *cb*_562_, the nanotube formation with Zn^2+^ was even induced^125^ and the presence of external amino acids that allowed coordination to the metal ions resulted in the formation of 2D nanoarrays.^126^,^127^

Triangular assemblies were directed by the complexation of Ni^2+^ ions to proteins embedded with recognition motifs such as 1,10-phenanthroline (Fig. 7c),^122^ attached covalently onto the surface of cytochrome *cb*_562_^6^,^122^ through the Cys59 residue located at the third helix of cytochrome *cb*_562_. Ni^2+^ is coordinated by a 1,10-phenantroline group at one protein monomer and a nitrogen of a His77 residue at another monomer.^122^ The triangular assemblies were packed into tubular units, and the superposition of different trimer orientations generated an apparent hexagonal hollow geometry.^122^ This was in contrast to the dimeric structures obtained from the examples discussed above,^79^ indicating that the interplay between different metal SLs and recognition motifs could play a strong role in PNs formation.

However, it was found in some cases that electrostatic interactions may play a more important role on the assembly formation than the metal coordination and the prediction of the protein nanostructure is not always straightforward.^79^ By selection of the metal–ligand interaction, light responsive protein PNs were prepared using GroEL.^83^ The biological role of GroEL in natural systems is to assist the refolding of denatured proteins by an adenosine-5′-triphosphate (ATP)-induced mechanism, which subsequently releases guest proteins.^128^ The barrel-shaped tetradecameric GroEL protein was first modified in the outer part of its cavity with a number of photochromic units, spiropyran/merocyanine, through 14 Cys residues spatially engineered on the top and bottom of the monomeric protein cylinder (Fig. 7d).^83^ The modified GroEL monomers then coordinated to Mg^2+^ in a light dependent fashion^83^,^129^ to form nanotubes with defined sizes.^83^ Other divalent cations such as Ca^2+^, Mn^2+^, Co^2+^ and Zn^2+^ were also able to induce assembly on GroEL-spiropyran/merocyanine, whereas monovalent cations (Na^+^, K^+^ and Cs^+^) were ineffective.^83^ Trivalent cations (Fe^3+^, In^3+^, Ce^3+^, and Eu^3+^), on the other hand, generated poorly defined aggregates,^83^ suggesting that the assembly could be triggered by electrostatic interactions rather the coordination bonds.^83^,^129^

The careful selection of the complexing ligands and metal ions as SLs allowed the formation of PNs but the influence of secondary interactions such as electrostatic interactions in the assembly has to be considered carefully,^79^,^130^–^132^ and greater predictability is required, perhaps by involving computation methods. Nonetheless, the SLs based on the metal–ligand strategy offers great opportunities in terms of structural and functional diversity if exogenous metal ions such as Pt^2+^ or Ru^2+^ are considered as well and the possibility to form a dynamic system by the interplay of metal SLs/ligand binding strength, which have mostly been neglected to date.

#### 2.2.5 SLs based on deoxyribonucleic acid (DNA) nanotechnology

**Fig. 8:**
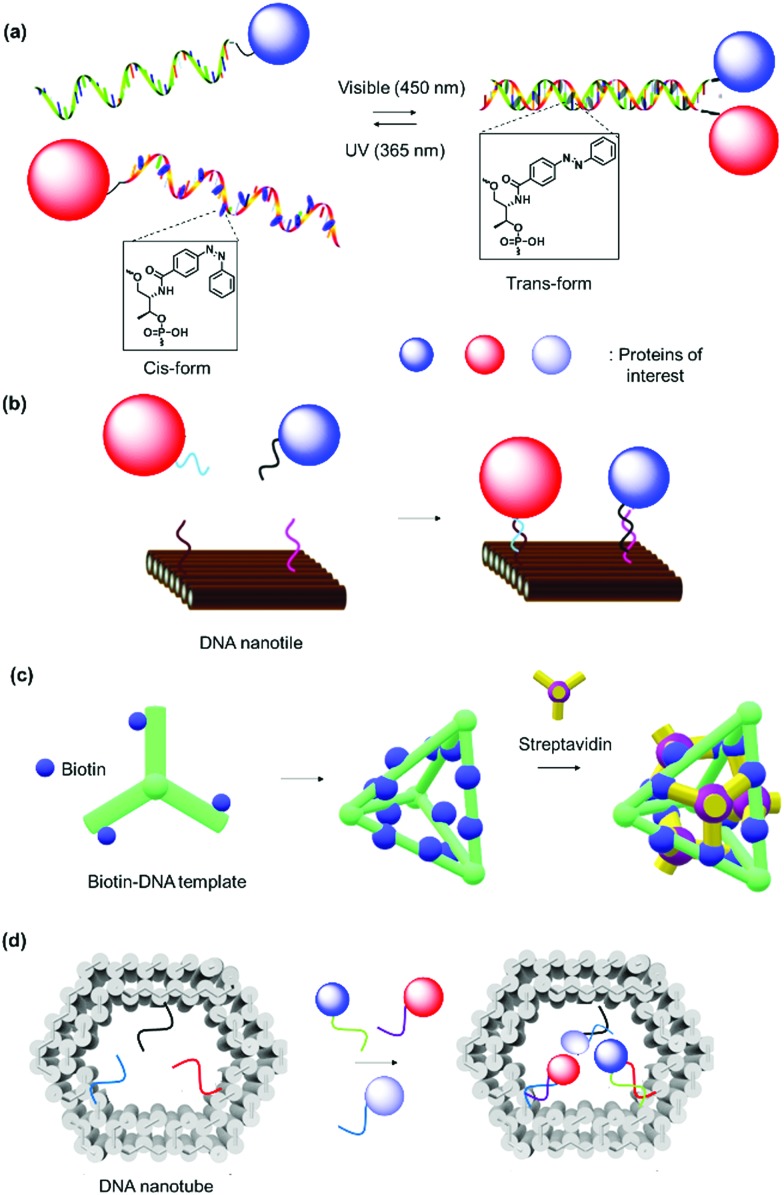
1D, 2D and 3D-DNA SLs. (a) Hybridization of ssDNA with complementary strand. The introduction of azobenzene in the oligonucleotide sequence allowed hybridization to be controlled *via* photoswitch. Adapted with permission from ref. 146. Copyright 2011 American Chemical Society. (b) DNA nanotile for the precise placement of proteins with defined interspatial distance. Adapted with permission from ref. 209. Copyright 2012 American Chemical Society. (c and d) DNA nanostructures as SLs for organization of proteins in 3D space. Adapted with permission from ref. 150 and 205. Copyright 2012 Wiley-VCH Verlag GmbH & Co. KGaA, Weinheim, 2015 Royal Society of Chemistry.

In nature, DNA plays an important role in directing protein assemblies. In coat proteins which are components that form virus capsids,^133^ the DNA cargo influences formation of the globular capsids. DNA nanotechnology has emerged as the method of choice to construct precise nanoscale architectures, due to the highly specific and predictable binding of complementary base pairs.^134^–^137^ The success of DNA nanotechnology has consequently led to an upsurge in research activities to exploit DNA as a template to arrange multiple proteins into well-defined nanostructures (Fig. 8).^138^,^139^ The DNA linker can be directly coupled to the protein of interest and then assembled onto the DNA scaffold. Alternatively, the protein of interest is attached directly to the DNA scaffold. Both approaches require either covalent or non-covalent conjugation of the desired PBs. Non-covalent methods include biotin–(strept)avidin, Ni^2+^–nitrilotriacetic acid, antibody–hapten, aptamer interactions.^140^ Here, aptamers are considered as shorter DNA sequences binding other compounds.^141^ Covalent strategies to connect proteins and DNA SLs include disulfide and maleimide coupling, protein ligation, biorthogonal click chemistry or enzyme-mediated reactions.^140^ DNA binding proteins such as zinc-finger proteins recognize specific DNA sequences, and they were fused to the desired recombinant proteins to guide self-assembly of proteins on DNA nanotemplates without additional chemical entities.^142^,^143^

Heterodimeric proteins were prepared by implementing complementary single stranded (ss) DNA sequences in the respective PBs (Fig. 8a)^144^–^146^ Interestingly, the spatial arrangement of DNA can be manipulated by applying the DNA origami method to obtain precise 2D or 3D templates, which allowed the self-organization of proteins in a convenient manner.^147^–^149^ By this method, a long “scaffold” strand DNA is molded into a desired shape “on demand” using hundreds of DNA “staple” strands (Fig. 8b). To achieve higher order nanostructures, a DNA polyhedral was prepared and in combination with biotinylation of a specific DNA strand to achieve trivalent binding on each of the polyhedral face.^150^ In this manner, proteins were organized on each face in 3D space with high spatial precision (Fig. 8c). Reverse engineering was also implemented by redesigning the binding specificity between DNA strands and coat proteins for the nanofabrication of PNs.^151^ Zlotnick *et al.* successfully demonstrated that the icosahedral viruses such as CCMV or cucumber mosaics virus can be reorganized to form uniform nanotubes and the length and diameter of the nanotubes controlled through the ratio of coat protein/DNA applied.^151^,^152^

Since the DNA staples are usually prepared synthetically, it was also possible to introduce molecular triggers in the design.^146^,^153^ Azobenzene was incorporated into the DNA linker and the resultant DNA duplex formation was controlled by light irradiation (Fig. 8a).^146^ In this fashion, the activity of the glucose oxidase/horseradish peroxidase cascade system was regulated by a light trigger, which offers unique opportunities in signal transduction controlled by external stimuli.^146^ DNA aptamers, which exhibit high affinity in the nanomolar range to a variety of proteins, were engineered into DNA tiles to target and direct the assembly of specific proteins such as thrombin or single-chain variable fragment (scFv) in a regular, periodic arrangement.^147^,^148^ Additionally, bidentate or tripodal tridentate aptamers were designed for the precise organization of proteins, as reported by Wilner *et al.*^154^ To improve stability of the DNA origami constructs, DNA linkers were replaced by peptide nucleic acids, where the phosphodiester backbone was replaced by a peptide backbone.^155^

DNA nanotechnology provides the possibility to tune the binding strength by the length or sequence of the DNA SLs (Table 2).^156^ For example, a 12-mer DNA consisting of adenosine only displays binding affinity with the complementary sequence that is three orders of magnitude lower than the 12-mer 5′-AGCTACACGATA-3 and comparable binding affinity to the 8-mer 5′-GCTACACG-3′.^156^ DNA nanotechnology can overcome some substantial challenges in terms of the precise alignment of individual protein components in multidimensions to form higher order nanostructures.^149^,^157^ Notably, it allows the exact number of proteins and interprotein distance to be predetermined by design. Significant advancements have been made in this field, and sophisticated nanoarchitectures and functions have been programmed, *e.g.* logic-gated nanorobots for targeted transport of molecular payloads such as antibodies^158^ or 3D multienzyme crystal structures,^74^ some of which are discussed in Section 2.3. Furthermore, DNA sequences are susceptible to cleavage by enzymes (DNAse) and this could confer responsiveness to the system.^159^ DNA nanotechnology was also combined with a protein backbone to enable multi-protein labeling by sequence-specific assembly through capitalizing on the unique features of DNA and protein materials.^160^ However, scale up and high cost production of DNA sequences as well as their highly negatively charged structures and low stability still limits this technology for several applications.^161^,^162^

The design of the SLs for specific interactions with recognition motifs discussed above certainly offer a broad spectrum of tools for nanofabrication of supramolecular protein architectures. It is obvious that the synthetic customization of PNs could be realized through rational selection of SL and recognition motif including electrostatic, protein–ligand and host–guest interactions, as well as metal coordination and DNA nanotechnology. Of all these strategies, DNA nanotechnology offer the highest structural precision, especially with recent breakthrough in the mass production of DNA origami.^163^ In addition, there is also parallel development of other synthetic methodologies to contribute to the growth of this emerging field, such as using nanoparticles,^164^ peptides,^157^ proteins,^165^,^166^ or polymers^167^,^168^ as templates to form PNs.

#### 2.2.6 Methods for quantification of SL–PB and PB–PB interactions

The SL–PB or PB–PB interactions and their assembly/disassembly control the stabilities of the PNs. Qualitative, indirect assessment could be through the “visual” observation of the PN formation and dissociation through imaging techniques such as atomic force microscopy or transmission electron microscopy. To gain greater insights into PN formation, quantitative analysis of the interactions of the recognition motifs on the PBs and the SLs are essential and they are typically determined in the form of enthalpy or binding constants. A number of biophysical methods, which are more sensitive, have emerged recently such as fluorescence spectroscopy, surface plasmon resonance (SPR), microscale thermophoresis (MST) and isothermal titration calorimetry (ITC). Some examples, as well as the advantages and limitations are described herein.

Fluorescence anisotropy/polarization (FP) is one of the common techniques used to unveil the relationship between proteins and their ligands in a quantitative manner due to the easy accessibility to the instrumentation.^169^,^170^ Fluorescence polarization determines the dissociation constant by measuring the rotational mobility of the ligand/protein before and after binding. It is a sensitive method and offers information on protein–ligand binding down to subnanomolar concentrations. Usually, a fluorescent ligand is titrated against varying concentrations of the PB to obtain a binding curve.^170^ Ross *et al.* used this approach to study the dimerization of endophilin, a 40 kDa SH3 domain-containing protein, with a dissociation constant of ∼5–15 μM in 20 mM HEPES buffer, pH 7.5, and 100 mM NaCl.^171^ Although less direct thermodynamic information can be obtained compared to ITC, the method requires lower amount of sample than SPR and ITC.^172^ The choice of a fluorescent ligand with a suitable lifetime that does not affect the SL–PB interaction is critical for accuracy.^170^ Moreover, the method is only applicable for ligands that are significantly smaller than the protein partner is and can only be applied outside cells. Non-fluorescent native ligands cannot be used directly for measurement and requires modification.

ITC is a sensitive calorimetric technique providing detailed information such as binding affinities and thermodynamic parameters of interacting biomolecules.^41^,^77^,^177^ Out of all the methods discussed in this section, ITC is a label-free method in which the heat evolved or absorbed during complex formation is measured through gradual titration of a ligand against the biomolecule of interest. It provides the affinity constant, stoichiometry, enthalpy and entropy of reversible biomolecular interactions.^41^,^77^,^177^ Quantification of the affinity range is from nanomolar to submicromolar. However, using competitive technique where the strong ligand displaces weak ligand–protein complex, dissociation constants within the picomolar range can be determined.^178^ ITC has been performed to elucidate thermodynamics parameters of the interactions of concanavalin A with SL consisting of α-d-mannopyranoside and rhodamine B.^41^ The resultant protein crystalline framework occurs through dual supramolecular interactions. Typically, protein crystallization is an entropy-driven process with a small change in enthalpy. But in this case, a negative heat of –63 ± 5 kJ mol^–1^ of SL was observed.^41^ The result is consistent with the sum of the binding enthalpies of concanavalin A with mannopyranoside and dimerization of rhodamine B (–67 kJ mol^–1^), thereby confirming the role of the SL to induce crystallization.^41^ Besides thermodynamic parameters, ITC analysis was employed to determine the ternary binding constant of CB[8]-(FGG-glutathione-*S*-transferase)_2_ as 2.9 × 10^12^ M^–2^,^179^ while a yellow fluorescent protein fused with FGG peptide displayed a ternary binding constant in the subpicomolar range.^77^ The major limitation of ITC is probably the need to dissolve all components in exactly the same solvent as well as low sensitivity towards a change in enthalpy. Therefore, solvents need to be selected carefully and investigations often require that one of the analytes is used in much higher concentrations compared to other techniques. This limitation could be circumvented by nano-ITC, which utilizes lower sample volumes (100 μL) and quantities (nanomole).

SPR measurements correlate the absorption of molecules on a thin, conducting surface ([Au] or [Ag] metal film) through the detection of changes in the refractive index at the surface of the film. SPR measurements give information on binding constants in nM to low mM range.^180^ Methyl-α-d-mannopyrannoside was immobilized to a thiol-modified gold surface to determine the dissociation constant to concanavalin A (∼μM range).^181^ Besides the dissociation constants, the on- and off-rate constants were also evaluated by SPR, for example, between a series of ribonucleic acids to a protein, NS3 protease domain of the hepatitis C virus.^182^ In comparison to the other methods, SPR is not conducted in solution and one of the binding partners has to be immobilized on the metal film, which could have an impact on protein structure and ligand binding. Consequently, it could be time consuming to establish a new assay, as site directed labeling of the PB needs to be optimized when different SL–PB or PB–PB interactions have to be investigated. Due to the limitations of mass transport close to the interface, SPR analysis could be complicated and surface immobilization could interfere with the binding event.

MST is a biophysical technique based on the motion of molecules in microscopic gradients and allows the determination of dissociation constant in the micromolar to picomolar range.^183^,^184^ The thermophoretic movement of the fluorescent molecules along temperature gradients triggered by an infrared laser on a sample in solution placed in a capillary and the mobility of the molecule is detected by fluorescence. The thermophoretic behavior is highly sensitive to variations in conformation, charge and size of the molecules due to a binding event and the binding affinity can be determined using a titration approach. Thermodynamic parameters can also be obtained by assessing the dissociation constants over a temperature range.^185^ These measurements can be carried out in minutes, with no limitation on molecular size, and they can be measured in buffer or complex biological media such as cell lysates and has low sample consumption compared to nano-ITC.^183^–^185^ MST measurement requires that one binding partner is fluorescent but it could also be conducted using the intrinsic protein UV-fluorescence. MST can record binding constants as low as the picomolar range, without the need of using competitive binding ligand like in ITC. However, the technique is sensitive to the presence of aggregates, provides less thermodynamic parameters compared to ITC and it is not suitable for studying weak interactions, *i.e.* in the mM range. Ng and Weil *et al.* investigated the facile assembly/disassembly of cytochrome *c*-polyethyleneglycol core–shell architecture formed *via* boronic acid-salicylhydroxamate interactions and determined the binding affinity to be in the micromolar range at physiological pH and dissociation at pH < 5.0.^186^ Similarly, Kuan and Weil *et al.* determined the pH dependent association and dissociation of a boronic acid modified lysozyme with a fluorescent dye consisting of salicylhydroxamate group.^63^

Besides the above biophysical techniques which measure the sample “bulk”, single-molecule methods, such as single molecule Förster (or fluorescence) resonance energy transfer (smFRET), have emerged that can resolve sample heterogeneity and thus allow probing of real time dynamics. smFRET has been successfully applied to investigate protein–ligand interactions at a single molecule level^173^,^174^ and even in living cells.^175^ It detects the non-radiative energy transfer between fluorescent donor–acceptor pair (<10 nm distance), which gives the intervening distance. In this way, binding rate and dissociation rates can be obtained, which is important for understanding biochemical processes in living cells since they usually occur under non-equilibrium conditions. smFRET can thus reveal more insights into molecular interactions, dynamics and mechanisms compared to other traditional biophysical methods.^174^ An additional advantage over other techniques is that smFRET allows investigation of binding with insoluble proteins. For instance, to study the dissociation rates (∼μM) of cell-bound T-cell antigen receptors binding to an antigenic peptide-major histocompatibility complex *in situ*. There are nevertheless some limitations to smFRET. It requires attachment of a minimum of two fluorophores to the analytes as the intrinsic fluorescence of tryptophan in proteins are not bright or photostable for measurement and weakly interacting fluorescent species could be challenging to study.^176^

With these analytical tools, quantitative information of PB–SL and PB–PB over a broad range of interaction strengths and stabilities could be obtained. Consequently, the results give valuable information for the optimization of the chemical design of PB and SLs for PN formation. It should be noted that the examples and the binding constants or thermodynamic parameters given above are based on established literature reports. They should only serve as a general guideline since binding constants and thermodynamic values could vary strongly under different conditions such as buffer used, ionic strength, temperature and pH.

### 2.3. Formation and characterization of the protein nanostructures (PNs)

In this section, the formation of PNs based on different permutations of PBs and SLs and the conditions in which they are formed are highlighted. The PN formation is discussed according to the resultant morphology, as well as their subsequent characterization. Typically, the formation of PNs is carried out in aqueous solutions such as phosphate buffer with variations in pH, buffer strength and additives, depending on the type of the PBs, SLs and recognition motifs used. Characterization with gel electrophoresis or size exclusion chromatography (SEC) reveal changes in retention time reflecting variations in molecular size.^187^,^188^ These are the most prevalent tools but they do not provide detailed information of the morphology of the PNs formed. In addition, it is very challenging to obtain insights on the precise ratio of PB units in heteromeric PNs. With the advancements in microscopy techniques such as atomic force microscopy (AFM),^189^,^190^ transmission electron microscopy (TEM),^191^ fluorescence correlation microscopy (FCS),^192^ as well as dynamic light scattering (DLS),^193^ more detailed characterization of PNs at the nanometer scale has been achieved. A summary of the PNs is given in Table 3.

**Table 3:** Overview of functional PNs

PBs	SLs/interaction motifs	Nanostructure (PN)	Application/function	Ref.
**Native PB**				
CCMV, avidin	Electrostatic surface patches	Binary crystals	Bioactive protein crystals	34
Apoferritin	Phthalocyanine	Crystals	Photosensitizer	90
SP1	CdTe quantum dots	Nanowires, nanorods	Light harvesting antenna	91
	Porphyrin-G5 PAMAM	Nanorods	Multienzyme cascade	92
Concanavalin A	Bismannopyranoside, mannose-rhodamine B	Protein crystalline frameworks	Porous protein material	41 and 42
Soybean agglutinin	*N*-Acetyl-α-galactosamine	Microtubule-like	Mimicry of microtubule	43
Streptavidin	Bis(biotin)–terpyridine	Polymer	Biomineralization	21

**Chemically modified PBs**				
Insulin	Bipyridine	Trimer	Regulation of glucose metabolism	65
Heme catalase	Oligonucleotides	Binary crystals	Cascade protein crystal reactor	74
Streptavidin-PAMAM, p53	Biotin	Dimer	Cytotoxic protein transporter	51
Avidin-HSA-PAMAM, β-galactosidase	Maleimide-biotin, iminobiotin	Trimer	Transporter of enzymatic protein cargo	20
Somatostatin, avidin, C3	Biotin–hydrazone-linker	Branched pentamer	Cancer cell-specific transporter, intracellular release	62
Antibody, avidin, LiDps cage	Biotin–streptavidin	Trimer	Selective uptake into *Staphylococcus aureus*	19
Her2, anti-CD3 antibodies, avidin	Biotin, Protein A–antibody	Pentamer	T cell-mediated lysis of Her2-positive breast cancer cells	199
Horseradish peroxidase, glucose oxidase	Oligonucleotides	Dimer	Enzymatic cascades	146
Anti-CD33 antibody; CDw238 FAb	3D DNA hexagonal barrel	Multimer in 3D scaffold	Nanorobot for stimulation of cellular processes	158

**Genetically modified PBs**				
CFP, YFP	CB[8]-Phe-Phe-Gly	Dimer	FRET pair	77 and 106
Split luciferase N- and C-terminal fragments	CB[8]-Phe-Phe-Gly	Dimer	Signal transduction with on–off switch	194
Cytochrome *cb*_562_	Bis(histidine)-Zn^II^	Tetramer; 2D protein array	Antimicrobial protein assembly in cells; template for nanoparticles growth	126, 127 and 229
Chaperonin GroEL, lactalalbumin	Spiropyran-Mg^II^	Nanotube	ATP induced release of cargos in HeLa cells	22 and 83
Glutathione-*S*-transferase	Histidine-Ni^II^	Linear or cyclic polymer	Catalytic elimination of cytotoxic compounds *in vitro*	84
	CB[8]-Phe-Phe-Gly	Linear or cyclic polymer	Inhibition of lipid peroxidation; nanospring	204 and 205
Hemeproteins	Heme	1D, 2D polymer	Oxygen transport	105
Cel5A, streptavidin	Biotin	Polymer	Biotemplating of artificial cellulosome	228
Streptavidin	Sortase A (G tag); horseradish peroxidase (Y tag)	Twigged polymer	Protein polymer scaffold for immobilization of multprtein	30

#### 2.3.1 Dimeric PNs

Protein dimers represent the simplest PNs and most functional examples in the literature consist of bi-enzyme cascades or combined fluorescence proteins, with the latter offering ease of characterization using fluorescence spectroscopy.^28^,^80^ Two identical protein copies interconnected by the SL form a homodimer,^28^,^80^ whereas the formation of heterodimers of two different proteins is more challenging as orthogonal interactions are required, and there is a more stringent demand on the PB and SL design.

**Fig. 9:**
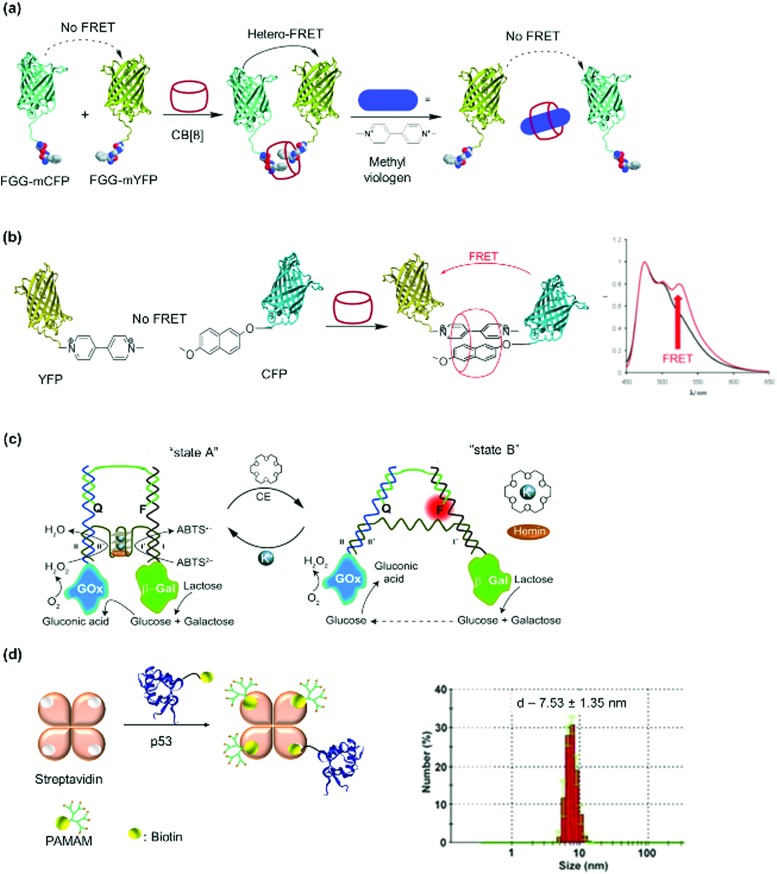
Dimeric PNs and characterization. (a and b) FRET fluorescence pairs formed using CB[8] host–guest interactions. Adapted with permission from ref. 77 and 106. Copyright 2010 Wiley-VCH Verlag GmbH & Co. KGaA, Weinheim, 2011 Royal Society of Chemistry. (c) Switchable enzyme cascade using DNA hybridization. Adapted with permission from ref. 197. 2014 Wiley-VCH Verlag GmbH & Co. KGaA, Weinheim (d) streptavidin bioconjugate formed by assembly of three PAMAM dendrimer branches (dendrons) and the respective cargo protein p53. Narrow size distribution was shown in DLS. Adapted with permission from ref. 51. 2013 Wiley-VCH Verlag GmbH & Co. KGaA, Weinheim.

A fluorescence/Förster resonance energy transfer (FRET) pair of Phe-Gly-Gly-CFP (cyan fluorescent protein) and Phe-Gly-Gly-YFP (yellow fluorescent protein) was prepared with the Phe-Gly-Gly peptide tag expressed on the N-termini of both proteins (Fig. 9a).^77^ Heterodimerization was induced in the presence of CB[8] in phosphate buffer at pH 7 and characterized by SEC. It was further substantiated by strong FRET, with an estimation that the protein heterodimerization was accompanied by about 50% homodimerization, which is reasonable since the same Phe-Gly-Gly tags were employed on both proteins. The addition of a small synthetic molecule such as methyl viologen (Fig. 6f), which is involved in competitive binding, resulted in the dissociation of the dimer.^77^ Similarly, two split fragments of the N-terminal (NFluc437) and C-terminal (CFluc398) of firefly luciferase were engineered with Phe-Gly-Gly peptides.^194^ In the presence of CB[8], the two non-active fragments were paired and luciferase activity was recovered. The formation of the ternary heterocomplex over the homocomplex was preferred due to higher stability of the former and confirmed by a titration against excess of the weakly binding Phe-Gly-Gly peptide.^194^ By using a supramolecular approach, an on–off switching mechanism was implemented by adding a competing ligand, such as the amantadine derivative 3,5-dimethyladamantan-1-amine (memantine), in conjunction with CB[8] for repeated up- and down-regulation of enzymatic activity, which is important for signal transduction.^194^

To circumvent the formation of homodimers as side products, orthogonal strategies are required. Besides the formation of 1 : 2 complexes with Phe-Gly-Gly peptides, CB[8] can also form stable 1 : 1 : 1 ternary complexes with an electron deficient–electron rich supramolecular guest pairs such as methyl viologen-alkoxynaphthalene to form a charge transfer complex.^106^ Brunsveld *et al.* successfully assembled alkoxynaphthalene-CFP and methylviologen-YFP FRET pairs using this approach (Fig. 9b).^106^ Through FRET measurements, they determined that the ternary approach using CB[8] eliminated the formation of homodimers.^106^ Split protein systems or protein heterodimers were also accomplished using DNA nanotechnology.^145^,^195^,^196^ For instance, switchable enzyme cascades such as glucose oxidase/β-galactosidase pairs with K^+^-ion stabilized hemin-G quadruplex horseradish peroxidase mimicking DNAzyme were prepared.^197^ First, the proteins were conjugated to two separate ssDNAs and the proteins dimerized on the complementary sequences on the template ssDNA (Fig. 9c).^197^ By using K^+^ ions and 18-crown-6, the template DNA switched between tweezers and clamp structures to activate and deactivate the catalytic activity.^197^ The occurrence of the reaction cascade to convert glucose to gluconic acid indicated that the bi-enzyme system was successfully prepared.^197^

Weil *et al.* first proposed a combinatorial approach where streptavidin was used as a supramolecular “glue” to fuse synthetic entities such as mono-biotinylated polyamidoamine (PAMAM) dendrons, with protein enzymes to mimic binary protein structures of AB-type bacterial toxins comprising of binding and catalytic domains.^51^,^198^ Here, the optimization of the biotinylated synthetic entity required for saturation of the binding pockets of streptavidin allows more precision in the stoichiometric loading of the cationic polyamidoamine (PAMAM) dendrons and the protein enzyme of interest, with high conjugation efficiency due to the strong streptavidin–biotin interactions. In this manner, the optimal stoichiometric ratio of the PAMAM dendrons and the protein enzymes, cytochrome *c* or tumor suppressor p53, were mixed with streptavidin at room temperature in phosphate buffer to give the tricomponent heterodimeric protein (Fig. 9d) and dendron-induced intracellular delivery of the protein cargo was successfully demonstrated.^51^,^198^ Dynamic light scattering (DLS) measurements supported the formation of the AB-type proteins with narrow size distribution (Fig. 9d). The method offers the advantage of rapid screening and optimization of the biological activity of a broad spectrum of biologically attractive combinations, as well as the possibility to fuse synthetic entities with biomolecules, which could not be accomplished by genetic techniques. However, this does not provide structural precision, especially if higher order nanostructures such as trimers or oligomers have to be achieved.

#### 2.3.2 Trimeric PNs

Fe^2+^ can interact with three units of bipyridine to form a six-coordinated octahedral complex. Capitalizing on this specific metal–ligand interaction, homotrimerization of PBs was achieved. A chemically modified PB, human insulin variant (InsX2) containing a single bipyridine, was added to Fe^2+^, and the complexation was confirmed due to a distinct colour change of the protein solution from colourless to magenta. A new ^1^H NMR signals of the bipyridine ligand appeared in the protein spectrum, and SEC showed a shorter retention time shift compared to the InsX2 monomer. The formation of a InsX2 trimer was corroborated in combination with static light scattering.^65^ Other homotrimers using metal–ligands are also discussed and referenced in Section 2.2.3.^122^

**Fig. 10:**
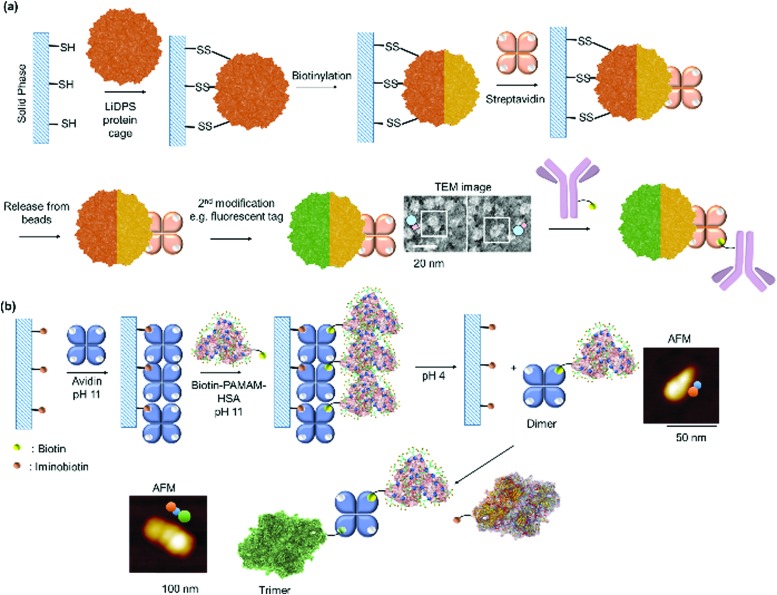
Solid phase approach for preparing heterotrimers based on (a) thiol solid support and (b) iminobiotin agarose and their subsequent characterization. Adapted with permission from ref. 19 and 20. Copyright 2009, 2013 American Chemical Society.

As mentioned earlier, the construction of heterodimers requires careful choice of PB and SLs and is challenging since homodimerization should be avoided. Thus, it is intuitive that the formation of trimeric or oligomeric heteroproteins would require much more synthetic efforts. Nevertheless, there has been some recent breakthrough with the development of solid phase preparation of protein nanoarchitectures.^19^,^20^,^199^ Solid phase protein synthesis approaches have been devised to desymmetrize homomeric protein building blocks such as (strept)avidin.^19^,^20^ Typically, these proteins possess more than one binding site but are symmetrical, and it is difficult to control the placement of different self-assembling protein components on these platforms. Solid phase approach was therefore devised to overcome this challenge.^19^,^20^ The solid phase exposes only one hemisphere of the protein building block and the other is masked and protected *e.g.* dynamic covalent S–S linkage or pH-sensitive non-covalent interactions (Fig. 10).^19^,^20^ In this manner, two-faced “Janus-like” PBs were derived to build up non-covalent heteroprotein nanostructures such as a heterotrimer.^19^,^20^,^198^ Notably, chemically post-modified PBs were applied to confer additional functions to the PNs, which could not be achieved by recombinant engineering such as the utilization of HSA incorporated with positively charged dendrons to enhance their cellular uptake.^200^

Douglas and co-workers developed a solid phase using disulfide linkage (Fig. 10a).^19^ A cysteine mutant of a protein cage, LiDps (DNA binding protein from *Listeria innocua*) was immobilized onto the thiol-functionalized solid phase in phosphate buffer at pH 7, followed by toposelective biotinylation of LiDPS, loading of streptavidin and release from the solid phase by reduction of the disulfide linkages with a dithiothreitol solution in sequential order generating a heterodimer platform.^19^ The heterodimeric structure was characterized by TEM, DLS, and quartz crystal microbalance. This nanoplatform was further coupled to biotinylated macromolecules such as antibodies.^19^ However, one possible drawback of this approach was that proteins, which are sensitive to redox conditions such as dithiothreitol cannot be applied as this could affect their activity.

Kuan and coworkers proposed a solid phase approach to desymmetrize avidin using the iminobiotin–avidin technology (Fig. 10b).^20^ Avidin was immobilized onto commercially available iminobiotin-agarose at pH 11, which effectively masked and protected one hemisphere of the avidin linker. Thereafter, a chemically post-modified protein conjugate, functionalized with a single biotin group (Fig. 3a) and PAMAM dendrons (DHSA) to enable cellular uptake, was anchored onto the available binding pocket of avidin. Subsequently, the heterodimeric DHSA–avidin conjugate was released from the solid phase by acidification (pH 4) due to protonation of iminobiotin, which abolished binding to avidin. The thus-prepared heteroprotein dimer still consisted of free binding pockets on avidin, which were used for further conjugation to other (imino)biotinylated molecule of interest, *e.g.* enzymatic proteins such as β-galactosidase and toxin enzymes.^20^,^198^ Fluorescence polarization is influenced by changes in molecular weight/hydrodynamic radius, thus affecting molecular mobility.^201^ The successful assembly of the heterotrimer was therefore confirmed by fluorescence polarization and the formation of a discrete trimeric protein structure was verified using AFM (Fig. 10b).

**Fig. 11:**
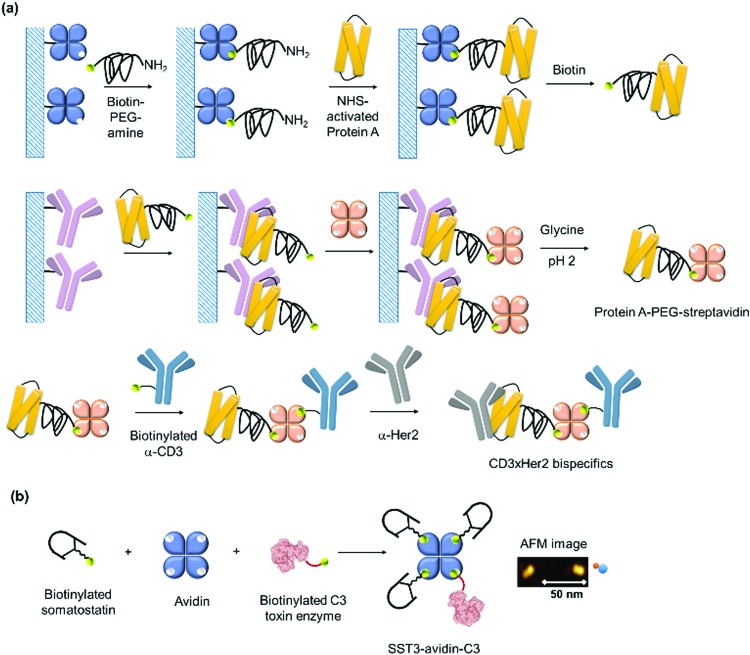
(a) Double solid phases approach for preparing heteropentamers based on biotin–avidin and protein/antibody interactions. Adapted with permission from ref. 199. Copyright 2014 American Chemical Society. (b) Nanoscale assembly of branched oligomeric SST3-avidin-C3 and AFM characterization of the PN. Adapted with permission from ref. 62. Copyright 2018 Wiley-VCH Verlag GmbH & Co. KGaA, Weinheim.

#### 2.3.3 Oligomeric PNs

Protein homotetramers have been reported using CB[8] as SL with a dimerizing YFP variant and N-terminal Phe-Gly-Gly as the PB. The formation of tetramers in phosphate buffer at pH 7 was characterized by a decrease in fluorescence anisotropy due to homo-FRET and a size increase in hydrodynamic radius from 3 to 4 nm in DLS measurements, as well as SEC.^78^

Homotetramers were also reported using SL based on metal–ligand interactions.^79^,^125^,^130^,^202^,^203^ For instance, a 4-helix bundle cytochrome *cb*_562_ PB with two sets of His dimers at the *i* and *i* + 4 positions formed tetramers upon addition of Zn^2+^ ions at higher concentrations. At lower concentrations, only monomers and dimers were observed, according to sedimentation velocity measurements,^79^ a method used to determine molecular size and shapes based on the rate of molecular movement according to the centrifugal force generated in an ultracentrifuge.^79^

Moving forward from homo- to hetero-oligomers, a solid phase approach was again implemented. Protein A, G, and L are surface proteins that are present in the cell walls of different bacteria and they exhibit high affinities and specificities to Fc region of immunoglobulins found in mammalian species, especially immunoglobin Gs. Using a dual solid phase approach based on Protein A/immunoglobulin G and biotin/(strept)avidin non-covalent interactions, Gao *et al.* prepared a Protein A–PEG–streptavidin heterobifunctional adaptor in a defined 1 : 1 : 1 stoichiometry, which was then used to prepare pentameric heteroprotein complexes (Fig. 11a).^199^ First, monomeric avidin resin was loaded with biotin–PEG–amine, followed by addition of NHS-activated Protein A to form monovalent Protein A–PEG–biotin conjugates. After elution of the Protein A–PEG–biotin conjugate from the column, streptavidin was added to Protein A–PEG–biotin immobilized onto a human immunoglobulin G agarose column. The tripartite 1 : 1 : 1 Protein A–PEG–streptavidin adaptor was isolated after elution from the column. Protein A can also be replaced by Protein L or G to expand the library of the available trimeric adaptor. This adaptor was characterized using gel electrophoresis, and it was further functionalized on its two ends with bioactive components.^199^ Notably, the authors prepared a hetero-pentameric complex where two antibodies were further conjugated to the Protein A–PEG–streptavidin adaptor. The successful conjugation was characterized by the preservation of both antibodies’ functions through the antibody-mediated uptake into on Her2-positive human breast cancer cells (SKBR3) and CD3-positive human peripheral blood mononuclear cells.^199^

Branched, oligomeric PNs were reported using the combinatorial approach discussed in the above section. Weil and coworkers constructed a branched, oligomeric polypeptide/protein nanostructure for cell type selective protein delivery.^62^ Three copies of the chemically modified cyclic somatostatin peptide hormone (SST) comprising a single biotin (Fig. 3b) were fused to avidin and the enzyme toxin C3 from *Clostridium botulinium*.^62^ The formation of the complex, SST3-avidin-C3 was carried out in HEPES buffer. AFM revealed a dimeric structure due to the larger avidin and C3 proteins as the somatostatin was too small to be detected by AFM (Fig. 11b). The complex formation was further corroborated by sodium dodecyl sulfate polyacrylamide gel electrophoresis (SDS-PAGE). The SST3-avidin-C3 was found to be stable in pH 7 buffer and human serum and allowed detailed *in vitro* and *in vivo* studies as discussed in Section 3.1. By employing a biotin SL with a pH-cleavable hydrazone linkage in the SST3-avidin-C3, the irreversible dissociation of the PNs was achieved in acidic conditions.

#### 2.3.4 Polymeric PNs

**Fig. 12:**
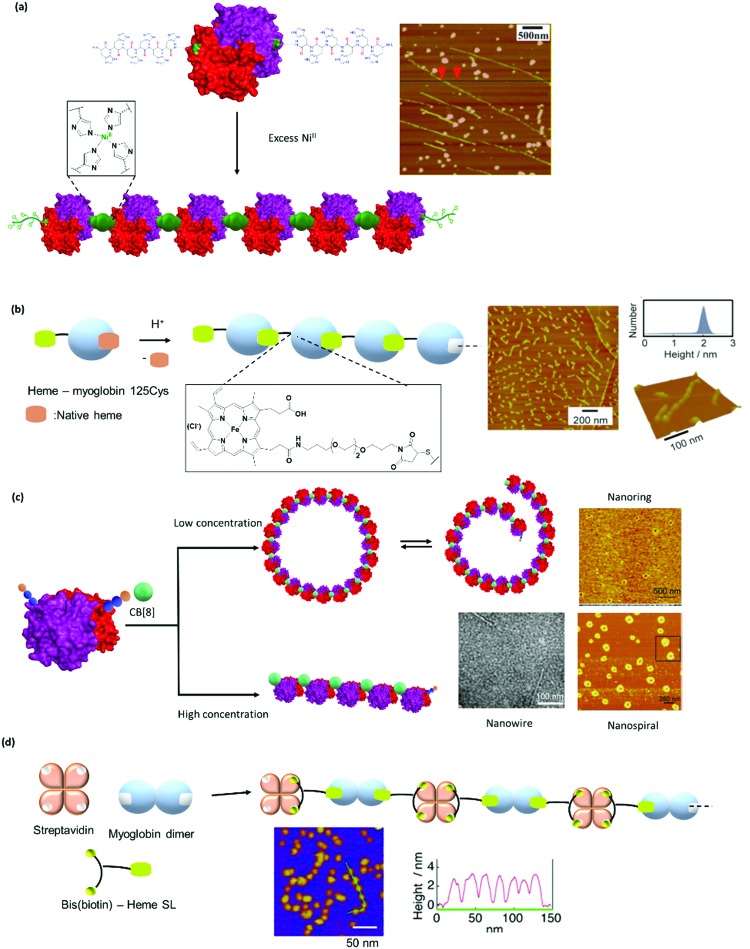
Formation of linear and non-linear supramolecular protein polymers. (a) Linear glutathione-*S*-transferase polymers through histidine-nickel coordination. Adapted with permission from ref. 84. Copyright 2012 Royal Society of Chemistry. (b) Myoglobin polymers from maleimide-modified myoglobin. Adapted with permission from ref. 75. Copyright 2011 Royal Society of Chemistry. (c) Nanoring PNs formed using recognition motif aligned in “V” arrangement and control of PB concentration. Adapted with permission from ref. 29. Copyright 2017 Royal Society of Chemistry. (d) Formation of A_2_B supramolecular protein polymer from streptavidin, myoglobin dimer and a bifunctional SL. AFM images of the PNs were shown. Adapted with permission from ref. 105. Copyright 2013 Wiley-VCH Verlag GmbH & Co. KGaA, Weinheim.

Linear supramolecular polymeric PNs have been reported with various combination of PBs and SLs including metal–ligand coordination,^84^ host–guest interactions^204^ and protein–ligand interactions.^105^ For instance, the generation of protein nanowires was achieved by using glutathione-*S*-transferase (GST) dimer from *Schistosoma japonicum* with a hexahistidine tag inserted at the N-terminus of each monomer.^84^ The localization of poly-His tags at opposite directions relative to each other allowed the formation of 1D-nanowires upon their coordination to Ni^2+^ using Tris buffer at pH 7.4, where one metal ion was coordinated by two terminal polyhistidine moieties at each end of two different GST dimers (Fig. 12a).^84^ The presence of Ni^2+^ SL was shown to be pivotal for the formation of nanowires since, in the presence of EDTA, the assemblies were reversed to the homodimers.^84^ The nanowires were characterized using AFM and gel electrophoresis. AFM measurements indicated objects with uniform heights of about 4.9 nm, consistent with the height of a single GST PB. Interestingly, a planar network was obtained by increasing the protein concentration through linear assembly, but this network did not display uniform heights. Native gel electrophoresis showed a few protein bands distributed over a range of molecular weights, suggesting that polydisperse PNs with distributions of the polymer lengths were formed. The protein nanowires presented slightly higher enzymatic activity, being able to catalytically eliminate *in vitro* cytotoxic compounds such as 1-chloro-2,4-dinitrobenzene when compared to the catalytic capacity of the homodimeric PB.^84^ Similarly, GST protein nanowires were prepared using the supramolecular interaction of CB[8] with two tripeptide Phe-Gly-Gly fused to the N-termini of dimeric GST with a C2 symmetry (Fig. 4c).^204^ To achieve a functional assembly, a glutathione peroxidase mimic was prepared with a single site mutation of selenocysteine (Y6C). Remarkably, the nanowires exhibited 20% inhibition in lipid peroxidation compared to the monomer in a mitochondria oxidative stress assay.^204^ Other functional features were also introduced by selection of an appropriate genetically modified PB. For instance, a recoverin domain, which is an allosteric protein responsive to Ca^2+^ with an N-terminal Phe-Gly-Gly tag was fused to dimeric gluthathione-*S*-transferase.^205^ The resulting protein nanowire exhibited conformational changes between a contracted and an extended state in the presence of Ca^2+^, thus functioning like a nanospring.^205^

Hayashi and co-workers made use of the heme-hemeprotein interactions to generate linear supramolecular protein polymers using cytochrome and myoglobin.^27^,^76^,^96^ More recently, sperm whale myoglobin with a single cysteine mutation at the 125 position was prepared, and an external heme ligand was introduced by maleimide-thiol reaction (Fig. 12b).^75^ Following the previously established method, the solution was acidified to generate an apo-myoglobin protein. Linear supramolecular protein polymers were achieved by switching the pH to neutral to induce interprotein heme–heme interactions, and the supramolecular PN was characterized by SEC and AFM.^75^ Interestingly, the myoglobin PN retained its bioactivity to transport oxygen.^75^ The addition of exogenous ligands such as CO or CN, which assume the axial position of the heme, could regulate the stability of the resultant supramolecular PN. Furthermore, the authors noted that a 3D protein network was achieved by cross-linking through the tyrosine residues using H_2_O_2_ and this network was characterized by scanning electron microscopy.^75^

The combination of lectin A with a tetraglyco-SLs with a rigid calix[4]arene core (Fig. 6c) preorganized the PB so that a 1D filament network with defined branching points imposed upon by the defect in the geometry in the glycocluster core was obtained.^99^ On the other hand, 1D nanoribbon wires of lectin A were obtained by using SLs containing α-d-galactopyranoside and rhodamine B (Fig. 6d).^40^ Interestingly, the resultant nanostructures can be fine-tuned to form 2D nanosheets or even 3D structures by applying different lengths of the tethering ligands.^40^

Aside from linear protein polymers, the formation of other morphologies such as ring structures were also achieved by the selection of a PB with a suitable orientation. By using glutathione-*S*-transferase with the arrangement of a recognition motif (Phe-Gly-Gly) arranged in a “V” shape (Fig. 4c),^29^,^132^ the resultant morphology of the PN was controlled to form nanorings. Bis-histidine metal chelating sites were introduced into the GST dimer from *Schistosoma japonicum* and addition of Ni^2+^ ions induced the formation of nanorings, as determined by AFM.^132^ Nanorings with different diameters were obtained by variation of the ionic strength of the buffer used, suggesting that the assembly process was driven by both protein–metal coordination and protein–protein interactions.^132^ Conversely, CB[8] induced polymerization was also achieved with a GST engineered with Phe-Gly-Gly tag in a “V” arrangement (Fig. 4c and 12c).^29^ Due to the relatively large size of the CB[8] SL, the protein–protein interaction was reduced compared to the Ni^2+^-histidine system, and PN formation was controlled by the ring-chain mechanism, which was affected by protein concentration.^29^ Consequently, a nanoring was prepared at low protein concentrations, and the assembly was transformed into a linear morphology by increasing the applied protein concentrations. Notably, nanospirals were achieved when a high ratio of CB[8] was added, as imaged by AFM.^29^

By astute chemical design, a bifunctional SL comprising bis-biotin and heme can be applied sequentially to connect two different PB monomers, namely, a natural PB (B), streptavidin and a genetically modified PB (A_2_), apomyoglobin dimer in alternating arrangement, to obtain the (A_2_B)_*n*_ linear protein polymer in K^+^ phosphate buffer at pH 7.0 (Fig. 12d).^105^ The SL was first appended to apomyoglobin dimer formed by a disulfide bridge, followed by subsequent addition of streptavidin and the formation of a 1D copolymer analysed by SEC. The molecular weight distribution of the obtained protein copolymers is affected by the spacer length of the SL and a short spacer prevented the formation of large copolymers. The size distribution was also controlled by varying the apomyoglobin dimer to streptavidin ratio. A smaller copolymer with narrower size distribution was obtained when using higher ratio of apomyoglobin dimer which, presumably terminated the polymer growth. SEC traces showed that a dodecameric (A_2_B)_12_ was the largest copolymer that was achieved and the formation of the alternating copolymer was further confirmed by addition of a known disulfide reducing reagent in order to convert the polymer back into the ABA trimer, which was characterized by AFM.^105^ Interestingly, the protein copolymer retained the dioxygen binding function of the heme cofactor.^105^ This supramolecular polymerization process was thermodynamically controlled and increasing concentrations of the PBs led to the formation of larger one-dimensional heterotropic assemblies.^105^ Dual supramolecular interactions were adopted together with dynamic covalent linkage (S–S) in apomyoglobin dimers, and this could serve as versatile platform to tailor a functional heteroprotein PN structures, which could be reorganized using multiple triggers.

#### 2.3.5 2D and 3D PNs

Besides one dimensional PNs, the formation of 2D- and 3D-PNs is attractive since they are often employed in Nature for scaffolding, as nanovessels, or cell components.^2^,^206^,^207^ DNA nanotechnology is the most prevalent strategy but suffers from scalability and thus there were also alternative strategies proposed.^83^,^97^,^126^,^127^ In this section, we highlight some examples such as micelle-like structures,^97^ protein nanotubes,^83^ and 2D protein arrays.^126^,^127^

**Fig. 13:**
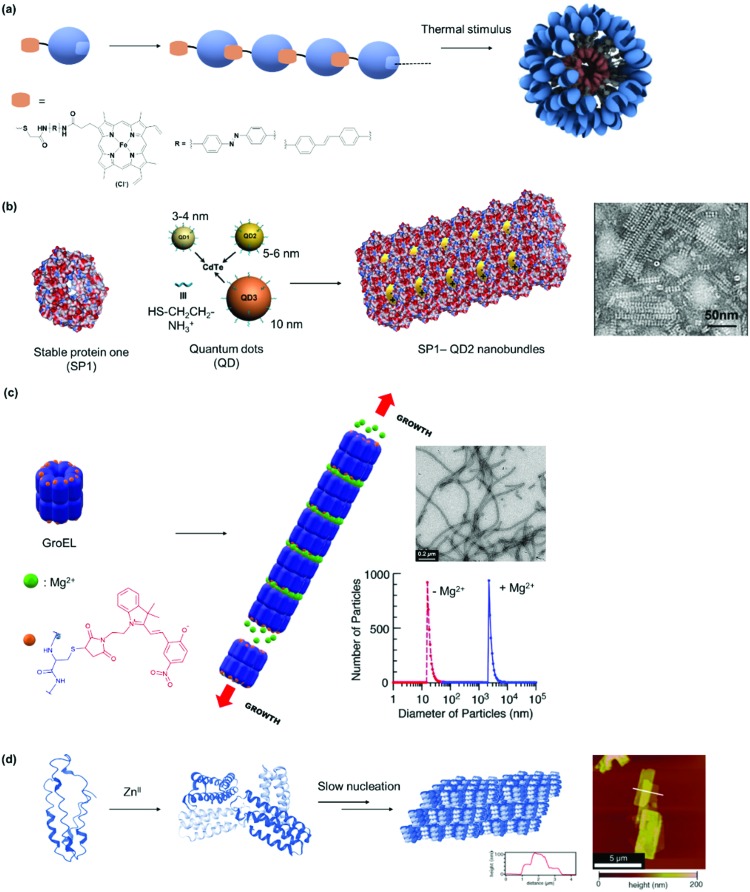
(a) Formation of micelle-type structures from linear hemeprotein polymeric PN driven by a transient thermal stimulus. Adapted with permission from ref. 97. Copyright 2017 Royal Society of Chemistry. (b) Nanobundles formed from charge driven assembly between cadmium/telluride quantum dots and stable protein one (SP1) as seen in TEM image. Adapted with permission from ref. 91. Copyright 2014 American Chemical Society. (c) Soft protein nanotubes formed by supramolecular polymerization of chaperonin GroEL. Characterization by TEM and DLS were shown. Adapted with permission from ref. 83. Copyright 2009 American Chemical Society. (d) Formation of 2D crystalline arrays from Zn^2+^ driven assembly of cytochrome *cb*_562_ as shown in AFM image. Adapted with permission from ref. 126 and 127. Copyright 2012 Nature Publishing Group, 2014 National Academy of Science.

Hayashi *et al.* installed an azobenzene or stilbenzene group in the design of their heme SL which served as a transient thermal stimulus to form thermoresponsive hemeprotein micelles (Fig. 13a).^97^ The heme SL was incorporated into a cytochrome *b*_562_ protein.^27^ SEC showed the formation of large assemblies, as previously observed for similar linear hemeprotein structures reported by the same group.^27^ Contrary to earlier examples, in which hemeprotein polymer dissociated into the monomer upon heating, a micelle-type structure was formed instead.^97^ The transitions between the morphologies were investigated *via* DLS and circular dichroism.^97^ The authors proposed that the switch could not have occurred *via* dissociation into the monomer but rather by the formation of a larger assembly, presumably a micelle, initiated by the denaturation of the protein at higher temperature (>80 °C), to eventually yield the kinetically trapped metastable micelle-type structure observed in TEM and DLS (diameter = 14–16 nm).^97^

By employing a cricoid PB such as stable protein one (SP1), charged induced assembly was initiated to form nanostructures ranging from nanowires, subsequent bundles, and irregular networks in aqueous solution.^91^ For example, nanobundles were obtained by selecting the right dimension of positively charged, globular quantum dots, as shown in AFM (Fig. 13b).^91^ Notably, the regular arrangement in the supramolecular structures allowed high efficiency (up to 99%) energy transfer, thus mimicking a light harvesting antenna.^91^ Nevertheless, a multienzyme-cooperative antioxidative system was created using this SP1 by incorporating selenocysteine to the PB and manganese porphyrin to the fifth generation polyamidoamine (G5 PAMAM), which was used a positively charged SL for self-assembly.^92^ In this manner, the SP1 acts as a glutathione peroxidase mimic while G5 PAMAM serves as superoxide dismutase mimic. The enzymatic cascade exhibits significantly enhanced biological activity over the individual components. SP1/core cross-linked micelle complexes self-assembled to form nanowires of more than 120 nm in length, where the extent of growth was adjusted by the electrolyte strength.^35^ At higher concentration, bilayered or trilayered large-scale nanorods were formed through staggering of the protein nanowires. Mimicry of the energy transfer process in natural photosynthetic bacteria *in vitro* was achieved where donor and acceptor chromophores were attached to stable protein one and spherical micelles, respectively, to obtain an overall energy transfer of 52%.

A protein-based soft nanotube was formed by supramolecular polymerization of the molecular chaperone GroEL through metal–ligand interactions (Fig. 13c).^83^ The barrel-shaped tetradecameric protein assembly was obtained in pH 7.4 Tris–HCl buffer by Mg^2+^-directed self-assembly in which GroEL was first spatially site-modified on the top and on the bottom of the cylindrical shape protein with a number of photocromic units, spiropyran/merocyanine.^83^ Monomers of GroEL-spiropyran/merocyanine coordinated to Mg^2+^ formed cylindrical fibers with a uniform diameter of 15 nm as shown by TEM.^83^ Interestingly, GroEL PBs were shown to not polymerize in the presence of Mg^2+^ suggesting that the photochromic units in spiropyran/merocyanine played a significant role in the nanotube assembly.^83^ The dependence of Mg^2+^ onto the formation of the assembly was attested upon the addition of EDTA. In the presence of this strong chelator, the cylinders were cut into short chain oligomers as well as into monomeric GroEL-spiropyran/merocyanine PB.^83^ Additionally, the formation of the nanotube was light-dependent and responded to mechanical force generated by adenosine-5′-triphosphate (ATP).^22^,^83^ Furthermore, GroEL-spiropyran/merocyanine presented similar binding affinity towards denaturated proteins, such as lactalbumin, when compared to native GroEL.^83^ To control nanotube growth, an engineered half-cut GroEL variant that firmly bound to the nanotube termini for end-capping can be employed.^123^ By variation of the end-capper to PB ratio, protein nanotubes ranging from 40 to 320 nm were obtained in a controlled manner.^123^ A DNA cleavable GroEL nanotube was reported by first replacing the metal-coordinating ligand with a ssDNA, *e.g.* 15-mer. Thereafter, a ssDNA incorporating the complementary sequence, *e.g.* 20-mer was used as a SL to induce supramolecular polymerization to form protein nanotubes in Tris–HCl buffer, as shown in TEM, DLS and SEC.^208^ The protein nanotubes were highly thermodynamically stable due to the multivalent interactions.^208^ Dissociation was achieved by adding an external ssDNA which consists of a full length complementary sequence to the 20-mer SL. In this way, the stronger DNA hybridization allowed the displacement of the SL, resulting in dissociation as observed in TEM, DLS and SEC.^208^

Cytochrome *cb*_562_ variants have also been employed for the formation of 2D-protein arrays. Tezcan *et al.* prepared Zn_8_PB_4_ units with cytochrome *cb*_562_ variant PB in which Cys and His residues were incorporated at positions 96 and 59, respectively.^125^ It was envisioned that His59 could contribute to Zn^2+^ coordination alongside with proteins’ natural weakly coordination domains such as glutamic acid, aspartic acid and alanine.^125^ The presence of Cys96 residue allows dimerization of the PB by Cys96–Cys96′ bridges to form PB_2_.^125^ The efficient formation of Zn_8_PB_4_ units was accomplished by the reaction between two units of PB_2_ and four equivalents of Zn^2+^ in the presence of Tris as a metal-coordinating buffer. The crystal structure of Zn_8_PB_4_ showed a 2D array in which it is possible to observe the formation of two sets of four internal Zn^2+^ complexes.^125^ The nanostructures resulting from Zn^2+^-directed self-assembly were shown to be dependent on time, Zn/PB ratio, and pH, as determined by TEM. Specifically, nanotubes were formed under high pH, high concentration of Zn/PB, and fast nucleation while the formation of 2D or 3D arrays was reported under low pH, low concentration of Zn/PB, and slow nucleation (Fig. 13d).^126^,^127^ The intermolecular interactions can also be tuned by chemical modification with a small molecule such as rhodamine B to induce crystalline arrays formation even under fast nucleation.^126^,^127^

**Fig. 14:**
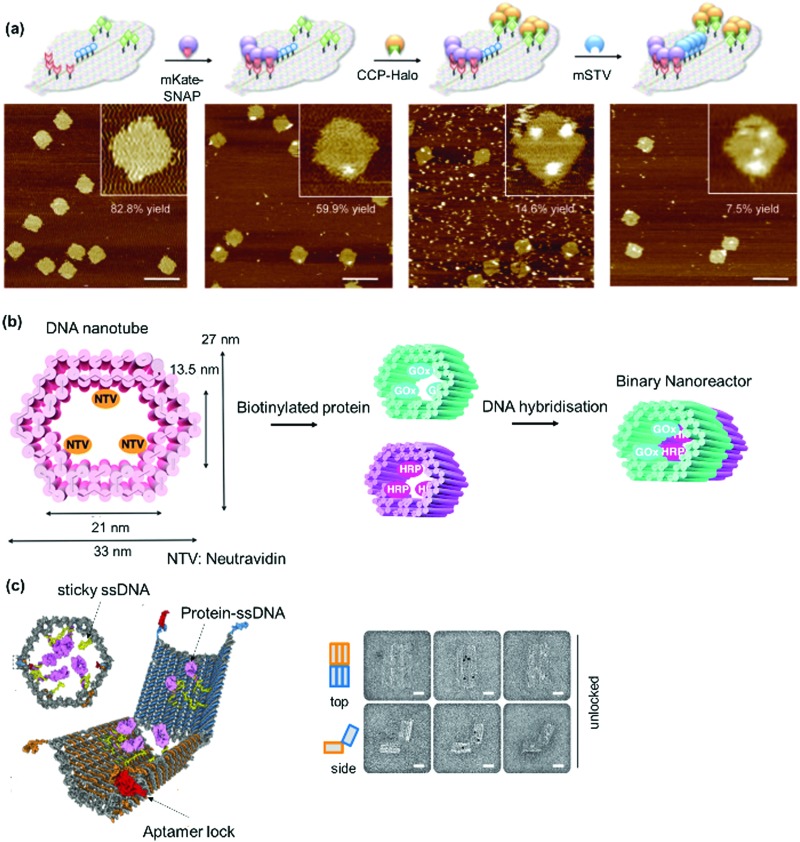
2D and 3D PNs formed using DNA nanotechnology. (a) Orthogonal decoration of proteins on 2D face-like DNA scaffold and AFM characterization of labelling yields at each step. Adapted with permission from ref. 138. Copyright 2010 Wiley-VCH Verlag GmbH & Co. KGaA, Weinheim. (b and c) DNA nanovessels for precise spatial placement of multiproteins in defined 3D arrangements. TEM image in (c) showed the protein loaded on the 3D hexagonal barrel. Adapted with permission from ref. 158 and 210. Copyright 2012 The American Association for the Advancement of Science, 2015 Royal Society of Chemistry.

DNA nanotechnology has been exploited for precise organization in 2D- and 3D-protein assembly and the simplest arrangement are represented by bienzyme cascades assembled on DNA tiles,^149^,^209^ with straightforward characterization by AFM. Notably, the spatial distance in multiprotein systems can be tuned with a great degree of control to modulate spatial interactions between the different protein components.^209^ By combining DNA nanotechnology, biotin–streptavidin, Snap tag and Halo-tag chemistry, Niemeyer *et al.* functionalized DNA strands for orthogonal complex multiprotein assembly on a biomolecular template.^138^ To demonstrate their concept, a 2D DNA face-like scaffold was designed using a software to ensure the correct folding of the M13mp18 ssDNA using 236 staples and the scaffold was characterized using AFM. Twenty three staple strands were biotinylated to create the eyes, nose, and mouth on the 2D face-like template (Fig. 14a). Monovalent streptavidin was allowed to bind and the obtained facial features were determined by AFM analysis. To introduce multiproteins, three chlorohexane (twice), four biotin, and four benzylguanine containing staples were immobilized on the origami scaffold as the eyes, nose, and mouth, respectively. Sequential addition of the proteins, namely mKate-Snap; CCP-Halo, followed by monovalent streptavidin led to formation of the intended smiley face structure, with an overall 7.5% yield. Each step of the assembly was characterized using gel electrophoresis and AFM analysis. The yields obtained were calculated with respect to the total of defined structures from AFM images. Furthermore, both sides of the quasi-2D plane could be decorated.^138^

DNA nanocontainers were prepared to encapsulate proteins in a modular fashion inside the cavity of DNA nanostructure. A hollow 3D DNA tube was employed as a nanocontainer to anchor NeutrAvidin, a deglycosylated form of native avidin through biotinylated DNA staple sequence protruding in the cavity.^210^ In this way, biotinylated enzymes such as glucose oxidase or horseradish peroxidase were attached in the cavity through the NeutrAvidin binding sites (Fig. 14b).^210^ Distinct units consisting of different functional proteins were then stepwise stitched together through design of the DNA base pairing to create, for example, a nanoreactor using glucose oxidase/horseradish peroxidase as a proof-of-concept system.^210^ In addition, proteins were also organized into 3D origami nanostructures in a spatially precise manner by DNA hybridization. A hexagonal DNA barrel with dimensions of 35 × 35 × 45 nm was prepared using a 7308-base filamentous phage-derived scaffold strand with 196 oligonucleotide staple strands (Fig. 14c).^158^ Notably, a hinge opening mechanism can be implemented with two domains covalently attached in the rear and noncovalently clasped together in the front by DNA aptamer-based lock mechanism that responds to binding antigen for unlocking. PBs such as antibody fragment, FAb, can be premodified to attach a DNA recognition motif, *e.g.* on the 5′ end of a 15-base ssDNA and guided to the sites of interest in the inward ring of the barrel nanostructure through hybridization with the DNA SL consisting of staple strands with 3′-extensions in the complementary sequences. TEM analysis showed that three FAbs were loaded and the 3D nanostructure was subsequently used as nanorobot in cell biology,^158^ discussed in more detail in Section 3.

#### 2.3.6 Crystalline protein frameworks

**Fig. 15:**
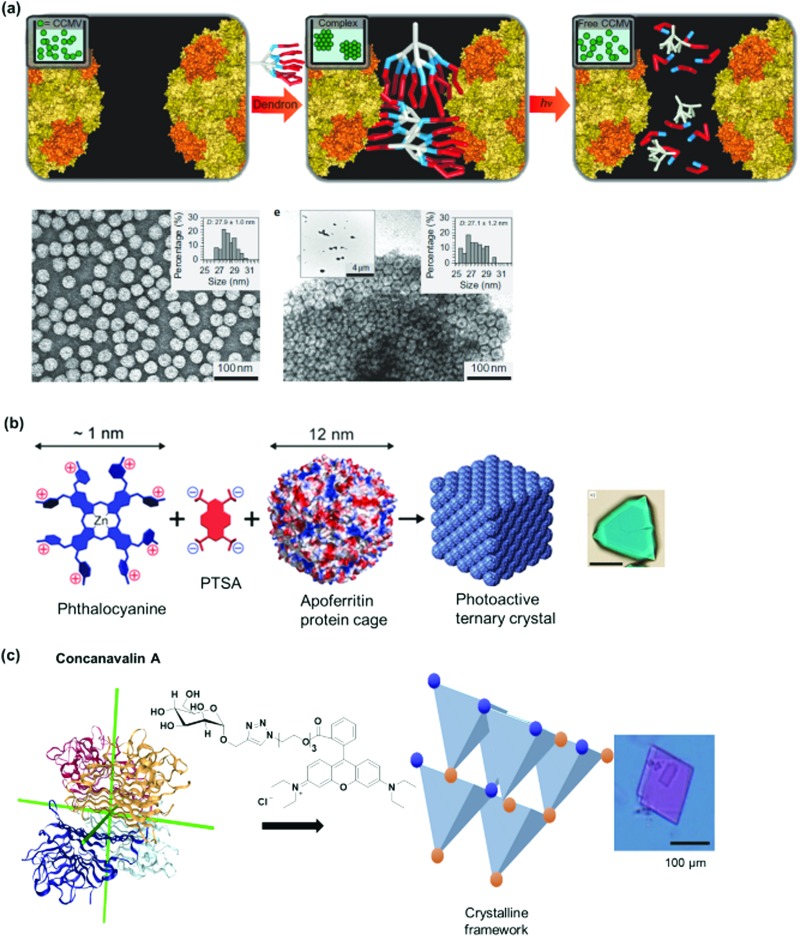
Protein crystalline frameworks. (a) Charge interactions of Newkome-type dendrons on CCMV surface patches and dissociation *via* photocleavage. TEM image of crystals are shown. Adapted with permission from ref. 88. Copyright 2010 Nature Publishing Group. (b) Photoactive ternary crystals formed from three-component systems with phthalocyanine. Adapted with permission from ref. 90. Copyright 2016 American Chemical Society. (c) Interpenetrating protein crystalline framework formed from concanavalin A and a dual interaction-SL. Adapted with permission from ref. 41. Copyright 2014 Nature Publishing Group. Optical images of the protein crystals in (b and c) were shown.

Single CCMV particles were assembled in aqueous solution into larger hierarchical structures based on multivalent electrostatic interactions with Newkome-type dendrons with a nitrobenzyl core (Fig. 5a).^88^ Both DLS and TEM investigations showed that the assembly was affected by the generation and concentration of the dendron as well as the ionic strength of the solution. The supramolecular architecture could be disassembled by the cleavage of the positively charged amine arms from the nitrobenzyl core by photolysis (Fig. 15a).^88^ To demonstrate the broad applicability, the assembly and disassembly of the dendron with a negatively charged protein cage magnetoferritin was also accomplished. Subsequently, Kostiainen *et al.* reported the ternary face-centered cubic (fcc) packed cocrystals of apoferritin and a supramolecular complex of octacationic zinc phthalocyanine and a tetraanionic pyrene formed by electrostatic and π–π interactions (Fig. 15b).^90^ The resultant structure preserved the fluorescent and singlet oxygen production properties.^90^ In this way, the resultant PN was able to produce singlet oxygen with a high quantum yield (0.72) upon irradiation, which is valuable for photodynamic therapy.^90^

Concanavalin A in combination with bismannopyranoside SL (red inset, Fig. 2c) formed a diamond-like protein lattices.^42^ The three-dimensional crystalline array was confirmed with X-ray diffraction, as well as TEM which show distances of 6.9 nm between molecules of the PB. A protein crystalline framework was also prepared using concanavalin A and the SL with dual molecular interactions consisting of α-d-mannose and a rhodamine B (Fig. 15c).^41^ Interestingly, the degree of interpenetrability of the crystalline framework was tailored by the linker design. The resultant concanavalin A protein crystalline framework was characterized by X-ray crystallography which showed that the mannose-rhodamine B SL binds to each of the four monomers of concanavalin A in a 1 : 1 binding.^41^ AFM images further revealed a square crystal with flat surfaces and sharp edges with a height of 200 nm, which was also substantiated by TEM.^41^ In these assemblies, the crystallization process was mainly driven by entropy, *i.e.* the protein–sugar binding occurred first, followed by rhodamine B dimerization to drive the entire self-assembly process to completion. Calorimetric measurements, circular dichroism and fluorescence lifetime measurements were employed to determine the kinetics and mechanism of protein crystal formation.^41^

Clearly, the combination of suitable PBs with SLs and recognition motifs allowed the synthetic customization of structurally defined, supramolecular PNs. In several instances, it was shown that the innate activities of the PBs are maintained or additional functions can be incorporated through appropriate chemical design of the SL. With the toolbox to engineer a variety of functional PNs in hand, the next step is to explore the applications of these synthetic PNs.

## 3. Functional supramolecular protein nanostructures (PNs) and their applications

In this section, the applications of PNs are discussed highlighting the functional activity of the resultant PNs and potential applications, together with future perspectives of these exciting new materials.

### 3.1. PNs for protein delivery into cells

Enzymes are emerging candidates for molecular targeting in diseased cells since they are usually highly specific in their mode of actions. However, applications are often limited by their low cellular uptake and proteolytic stability. The expression of fusion proteins consisting of enzymes and cell targeting entities such as antibodies (Abs), translocation domains of toxins and cell penetrating peptides (CPPs) has enabled cellular delivery of the enzyme cargo.^211^–^214^ Since the fusion takes place at the genetic level, it is not feasible to include chemically modified proteins or customize linkers equipped with various cleavage groups between the proteins in the design. Chemically engineered fusion proteins represent an attractive strategy to expand the repertoire of natural or recombinant fusion proteins particularly for the incorporation of post-modified proteins. Linker groups with pH- or light-cleavable groups allow the controlled release of the protein cargo in the microenvironment of diseased cells with external stimuli.^215^ In this context, dimeric and trimeric PNs have been prepared consisting of a pH cleavable linker connecting a transport protein with a cargo protein to achieve efficient delivery and controlled release of the protein cargo in cancer cells.^20^,^62^

**Fig. 16:**
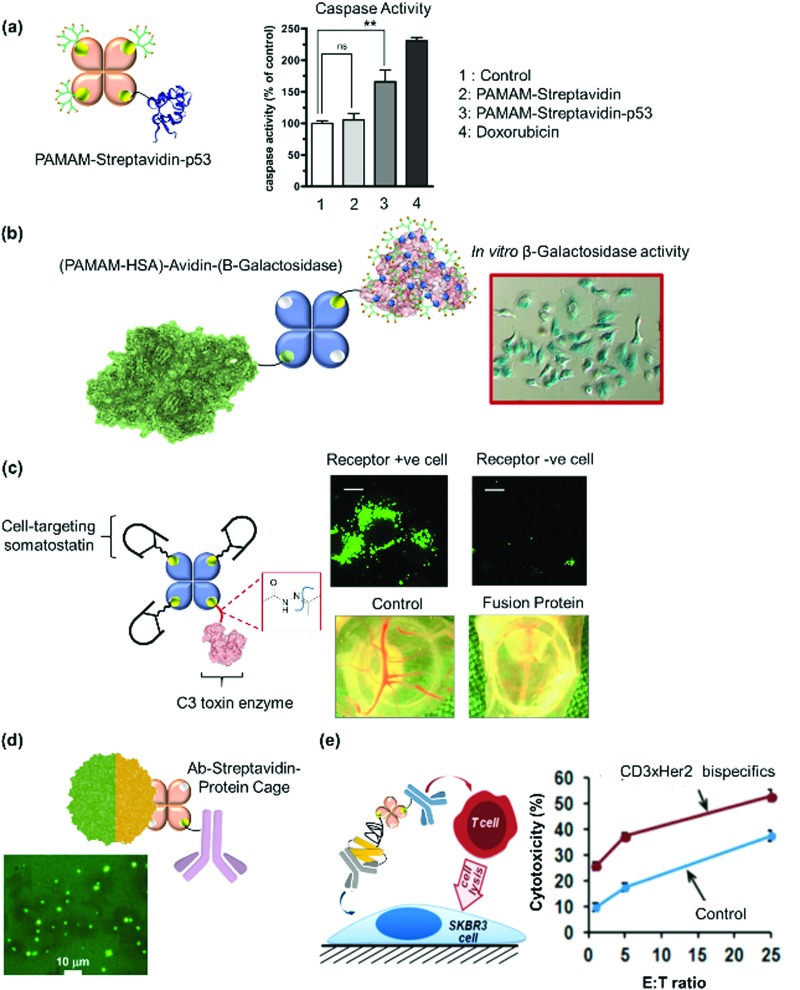
PNs for delivery of active proteins into cells. (a) Delivery of tumor suppressor p53 to induce apoptosis *via* Caspase 3/7 activation. Adapted with permission from ref. 51. 2014 Wiley-VCH Verlag GmbH & Co. KGaA, Weinheim. (b) Intracellular delivery of β-galactosidase into A549 cancer cells. The blue coloration of the cells showed that the enzyme preserves its bioactivity *in vitro*. Adapted with permission from ref. 20. Copyright 2013 American Chemical Society. (c) Selective delivery of C3 into cell lines overexpressing somatostatin receptors was shown compared to a receptor negative cell line. The application of the fusion protein in chick embryo chorioallantoic membrane model showed an antiangiogenic effect. Adapted with permission from ref. 62. Copyright 2018 Wiley-VCH Verlag GmbH & Co. KGaA, Weinheim. (d) The internalization of a fluorescently labelled LiDPS protein cage into the microbial pathogen, *Staphylococcus aureus*, was mediated by assembly with an antibody. Adapted with permission from ref. 19. Copyright 2009 American Chemical Society. (e) CD3xHer2 bispecific antibody target Her2-positive human breast cancer SKBR3 cells increase cytotoxicity due to T-cell mediated lysis. Adapted with permission from ref. 199. Copyright 2014 American Chemical Society.

Heterodimer proteins consisting of streptavidin containing a polyamidoamine (PAMAM) shell and a cargo protein have been assembled by the biotin–streptavidin technology. As cargos, biotinylated cytochrome *c*, the tumor suppressor protein p53^51^ or the C3 toxin, a specific Rho-A, B and C inhibitor, have been selected and connected to PAMAM-streptavidin.^198^ Both p53 and C3 are relevant for cancer therapy but they are not taken up by cells. The formed fusion proteins were internalized into A549 lung cancer cells by clathrin-mediated endocytosis due to the positively charged PAMAM dendrimer branches. The fusion protein consisting of PAMAM-streptavidin and p53 resulted in cell death *via* caspase 3/7 activation (Fig. 16a) whereas the heterodimer comprising the C3 toxin enzyme induced changes in cell morphology through inhibition of the Rho A protein.^51^,^198^

More sophisticated, trimeric fusion proteins have been achieved consisting of blood plasma protein HSA that was decorated with a PAMAM corona (HSA-PAMAM) to enable efficient uptake into mammalian cells such as A549 lung cancer and HeLa.^20^,^198^,^200^ As cargo, different protein enzymes such as C3 toxin, β-galactosidase and the enzymatic subunit of the *Clostridium botulinum* C2 toxin, C2I were assembled with HSA-PAMAM by an avidin (Av) linker. pH-controlled release of the cargo protein was achieved by conjugating the pH-sensitive iminobiotin to the cargo enzymes to enable cleavage of the enzyme cargo from the avidin carrier under acidic conditions.^20^ Notably, intracellular release of the cargo proteins under acidic endosomal conditions was demonstrated, and both the internalized C3 toxin and β-galactosidase preserved their enzymatic activity in cells (Fig. 16b). However, these chemical fusion proteins were not specific to cell-types and, given their potencies; it would be desirable to deliver the enzymes specifically into cancer cells in order to avoid adverse effects.

To impart cell-type selectivity, peptide targeting entities recognizing membrane receptors at the surface of cancer cells would be highly desirable.^216^,^217^ A structurally refined PN was assembled combining a central avidin decorated with a single C3 toxin cargo somatostatin and on average three copies of the peptide hormone somatostatin (Fig. 3b) that targets specific cancer cells such as lung carcinoma A549 that overexpresses the somatostatin receptors.^62^ As another level of structural complexity, a pH cleavable hydrazone group was introduced in the linker (Fig. 6e) interconnecting Av and C3 toxin to achieve cargo release in acidic intracellular compartments such as endosomes.^62^ By redirecting cell surface interactions, intracellular uptake and cytosolic release of a therapeutically relevant enzyme by three somatostatin peptides was obtained. This protein assembly showed antiangiogenic effects (Fig. 16c) and 100-fold improved potency compared to the therapeutic Ab Avastin in an *in vivo* chick embryo chorioallantoic membrane model of advanced non-small cell lung cancer. The PN was also applied in combination with the marketed chemotherapeutic drug doxorubicin, which enhanced the efficacy of doxorubicin by about three-fold, both *in vitro* and *in vivo*.^62^ These examples demonstrate the versatility of constructing customized protein assemblies with structures that could not be created with the cellular machinery and unique biological activities.

Protein cages are often employed as platforms for transport of therapeutics or imaging agents by loading the cargoes into their cavities. They are attractive for passive targeting whereby larger nanoparticles show preferential uptake through the leaky vasculature of the tumour microenvironment by the enhanced permeation and retention effect.^218^ However, the passive targeting effect is often not substantial and offers only moderate transport to cancer tissue and cells.^219^ Consequently, strategies to attach antibodies to address membrane receptors overexpressed at tumor cells have been developed to enhance their therapeutic efficacy. A protein trimer consisting of an anti-Protein A Ab connected *via* streptavidin to the DNA binding protein from *Listeria innocua* (LiDps) was prepared by solid phase synthesis.^19^ Flow cytometry clearly showed that the Ab fusion protein mediated the uptake of the LiDps cage into the microbial pathogen, *Staphylococcus aureus* (Fig. 16d), which was otherwise not possible.^19^

Moreover, the preparation of “bispecifics” that comprise two Abs or antigen binding fragments (FAb) able to simultaneously bind two different antigens have been introduced to develop highly specific and potent biotherapeutics.^220^ For example, Ab bispecifics are important for immunotherapy to direct T cells against cancer cells to eliminate tumors.^220^ Furthermore, the anti-CD3 (cluster of differentiation 3) Ab binds to CD3 on the surface of T cells and acts as immunosuppressive drugs to direct T cells against cancer cells. Both Abs, anti-Her2 and anti-CD3, were assembled on the Protein A–PEG–streptavidin adaptor described in Section 2.3.3. to form the bispecific Ab. Flow cytometry assays confirmed that the Ab bispecific construct was able to target both Her2-positive human breast cancer SKBR3 cells in combination with CD3-positive human peripheral blood mononuclear cells, which served as the effector cells.^199^ Therefore, the CD3xHer2 bispecific Ab had an effect on T cell-mediated lysis of Her2-positive breast cancer cells. It increased cytotoxicity by 15–20% compared to the control using a mixture of anti-CD3 and anti-Her2 (Fig. 16e), which could be attractive for tumor-targeted therapy.

### 3.2. PNs as nanorobots in biology

In Nature, a variety of biomolecular nanoscale devices comprising of proteins have been formed and are known to play vital roles in important functions *e.g.* cell division and signal transduction.^221^ Inspired by such natural nanomachines that act in a concerted fashion, Nobel Laureate Richard Feynman envisioned smart nanomachines that could respond to molecular cues in a cellular context to regulate biochemical process or effect changes in the cellular biochemistry.^222^,^223^ Consequently, synthetic PNs have been devised that could serve as smart nanorobots in a cellular context.^22^,^158^

**Fig. 17:**
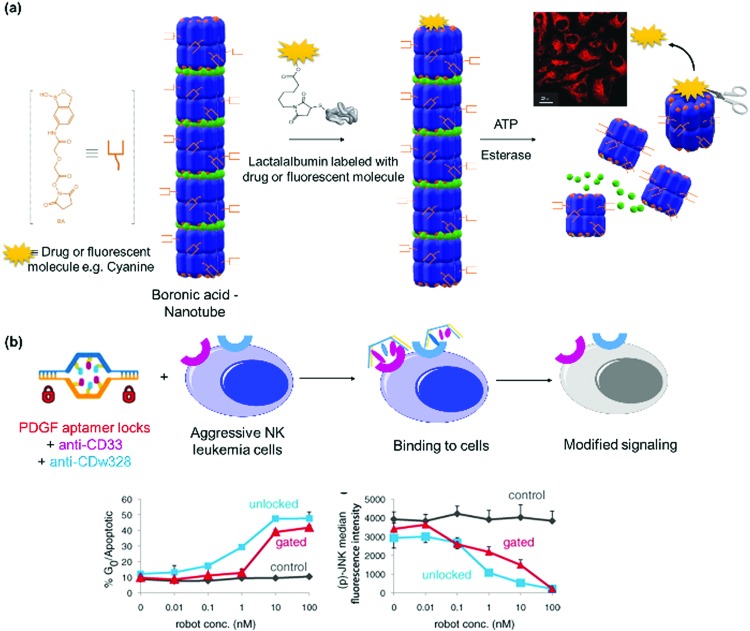
(a) Boronic acid-functionalized nanotubes deliver a dye conjugated to denatured lactalalbumin, which was released in the presence of ATP and esterases. The confocal image showed the uptake and release of the fluorescent cargo. Adapted with permission from ref. 22. Copyright 2013 Nature Publishing Group. (b) 3D nanorobot loaded with anti-CD33 and anti-CDw328 antibodies modified signaling in aggressive NK leukemia cells. The cell cycle distribution and phosphorylation level of Jun N-terminal kinase as a function of robot concentration after 72 hours was determined. Adapted with permission from ref. 158. Copyright 2012 The American Association for the Advancement of Science.

Cylindrical, tube-like PN have been developed as “nanorobots” for delivery and programmed to specifically release cargoes in response to a biological signal.^22^ Compared to spherical structures, protein tubes provide several attractive features as both ends of the tubes are open, allowing material exchange from the inside to the outside by simple diffusion. By capitalizing on the innate feature of GroEL to assist the refolding of denatured proteins^128^ and high ATP concentrations inside cells, which range from 1–10 mM,^224^ intracellular delivery and release of a fluorescent dye using a GroEL protein nanotube was shown. Aida and co-workers prepared a boronic acid modified GroEL protein nanotube, which was taken up into human epithelial carcinoma HeLa cells through preferential binding to the glycoproteins and glycolipids at the outer HeLa cell membranes.^22^ The nanotube was used to incorporate the fluorescent dye cyanine, bound to denatured lactalalbumin *via* an esterase-cleavable linkages (Fig. 17a). It was applied for the delivery and controlled release of imaging agent or even drug molecules, in HeLa cells, in the presence of ATP and esterase. Moreover, the nanotube preferentially accumulated in the tumor tissue when compared to other tissues, except liver tissues, which is very attractive for *in vivo* applications.^22^

A 3D nanorobot able to transport molecular protein cargoes, such as a combination of antigen-binding fragments (FAb), a region on an Ab that binds to antigens, and respond to a variety of molecular cues in the cellular context to regulate the offloading of the FAb cargoes has been realized using DNA nanotechnology.^158^ A lock was designed in the DNA barrel based on aptamer recognition (Section 2.3.5) where two simultaneous molecular events have to occur to activate the opening, giving rise to a logical AND gate. In this manner, combinations of different molecular cargoes such as FAb and aptamer lock could be implemented to tailor the delivery and release of the molecular payloads in a highly selective biological environment.^158^ For example, the molecular cargoes containing Abs against human CD33 and against human CDw238FAb′, which induce growth arrest in leukemic cells, were loaded with precise spatial organization in the hexagonal barrel nanostructure. A pair of aptamer sequence 41t against platelet-derived growth factor was implemented in the design to address large granular lymphocytic leukaemia, aggressive NK type (NKL) specifically.^158^ A dose-dependent induction growth arrest of NKL cells through the suppression of Jun N-terminal kinase and protein kinase signalling was observed (Fig. 17b). On the other hand, incubation of a nanorobot consisting of Ab against human CD3εFAb′ and Ab against flagellin FAb′ led to the recruitment of flagellin at low concentration (100 pg mL^–1^) and induce augmented T cell activation, which is a convenient tool to bring about changes in cellular behaviour in a controlled way.

### 3.3. PNs as templates

**Fig. 18:**
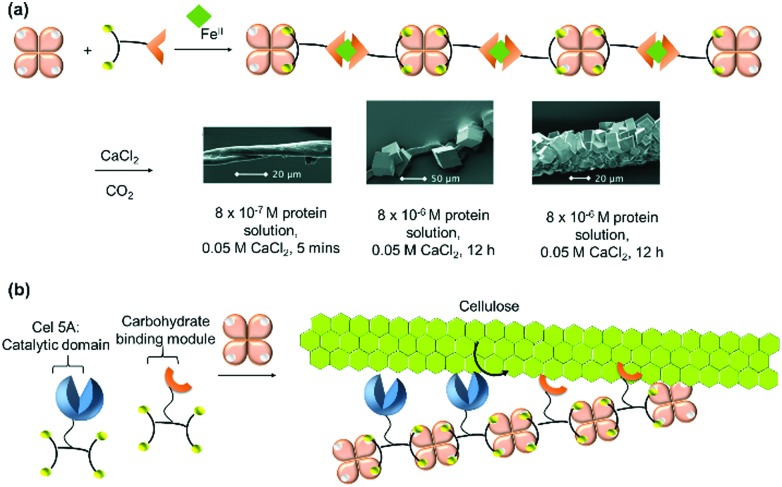
(a) Streptavidin-templated biomineralization of calcite microcrystals (from CaCl_2_ solution) over time. Adapted with permission from ref. 21. Copyright 2007 Wiley-VCH Verlag GmbH & Co. KGaA, Weinheim. (b) Streptavidin-templated polymerization of Cel5A, an endoglucanase, to create an artificial cellulosome, a multi-enzyme complex associated with cell surfaces. Adapted with permission from ref. 228. Copyright 2016 Royal Society of Chemistry.

Amyloid fibers consist of very stable β-sheet structures made up of self-assembling peptides. They are often employed in Nature as a synthetic template or as efficient storage modules.^225^ Based on this phenomenon, there have been various groups who used self-assembling peptide fibers as templates for nanoparticle growth, neuronal cell growth, stem cell differentiation,^226^ or for polymerization such as formation of poly(dopamine) on the surface of peptide fibers.^227^ However, there are not many examples known to date for PNs as biotemplates. One of such few examples includes using a linear streptavidin polymer as a template for biomineralization.^21^ The linear polymeric PN formed bundles with micrometer diameter and a millimeter length in CaCl_2_ solution, due to chelation to the aspartate and glutamate side on the surface of streptavidin. Biomineralization of calcite microcrystals was accomplished using the streptavidin PN as a template in the presence of ammonium carbonate as a carbon dioxide vapor source (Fig. 18a). This process mimics collagen processing with a hierarchical assembly from nanoscale PB to millimeter supramolecular structures.^21^

An artificial cellulosome was created using streptavidin–biotin polymerization as a template (Fig. 18b).^228^ Glycoside hydrolase family 5 (Cel5A) from *Thermobifida fusca* was selected as a model enzyme for biomass degradation to hydrolyse β-1,4-glycosidic bonds of internal cellulose chains in the amorphous region.^228^ The catalytic domain of the enzyme was fused with a microbial transglutaminase tag (Met-Arg-His-Lys-Gly-Ser; K-tag) and a tetravalent bis(bis(biotin-Gly-Gly-Gly)-Lys)-Lys-Gly-Leu-Gln-Gly ligand was introduced site specifically to interact with the cis dimer of streptavidin. To mimic the natural cellulosome system where different types of enzymes are recruited through cohesin–dockerin interactions to optimize the hydrolysis activity depending on the biomass type, a heteroprotein complex comprising of Cel5A with a K-tagged cellulose binding module (CBM) from *Bacillus halodurans* was co-assembled. The resultant supramolecular complexes, (Cel5A)_*n*_(CBM)_*m*_ – (streptavidin)_*n*+*m*_ demonstrated enhanced activity for saccharification compared to the self-assembled complexes of the individual components alone. Although the actual spatial order of the individual proteins in the 1D structure cannot be precisely controlled, the catalytic activity can be optimized by the loading ratio of the building blocks.

Tezcan and coworkers have developed 2D crystalline arrays based on Zn^2+^-coordinated cytochrome *cb*_562_ variant where the Fe heme cofactor is replaced by zinc porphyrin and used it for the growth of platinum nanoparticles.^127^ Although this has not been applied in a biological context, the same group has recently reported the possibility to perform the self-assembly of cytochrome *cb*_562_ in *Escherichia coli* cells^229^ and such system could hold immense promise to induce nanoparticle growth in living cells.

### 3.4. Constructing bioactive PNs in cells

Most of the PNs demonstrated are carried outside the cells but in Nature, protein assemblies occur naturally in cells to form nanostructures or architectures that support the essential functions of organisms such as compartmentalized structures like organelles and nanovessels for molecular storage or transport. Hence, there is a burgeoning interest to translate the various strategies to achieve assembly in living cells to come up with entirely new structural and functional features. In recent years, this has met with some success by constructing PNs using genetic tools^207^,^230^–^232^ or synthetic molecules such as N-heteroaromatic quaterthiophene analogues.^233^ For example, genetically encoded amphiphilic block-domain proteins were reported to form cellular compartments or organelles in *Escherichia coli*.^207^ By introduction of non-natural amino acids, they can be further functionalized with chemical functionalities such as a fluorescent dye to confer additional properties to the artificial self-assembled organelles.

**Fig. 19:**
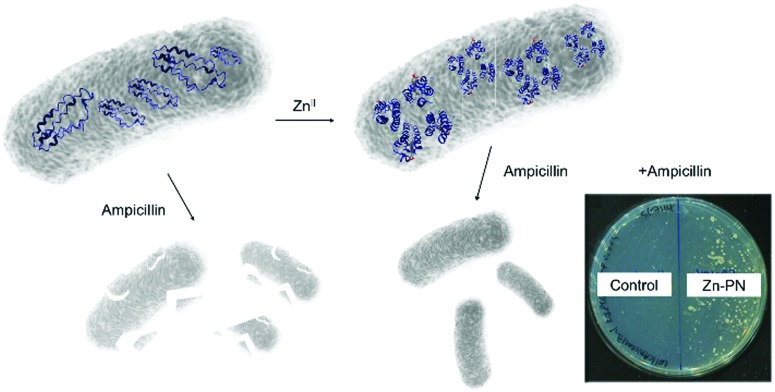
Self-assembly of tetrameric cytochrome *cb*_562_ variant in *E. coli* in presence of Zn^2+^ ions forming Zn-PN. The Zn-PN exhibited *in vivo* metallo-β-lactamase activity, even in the presence of ampicillin, a β-lactam antibacterial chemotherapeutic drug. Adapted with permission from ref. 229. Copyright 2014 The American Association for the Advancement of Science.

However, there are far less examples to construct PNs prepared in cells using synthetic approaches discussed herein. The only example until now involves an artificial metallo-β-lactamase based on the Zn_8_PB_4_ motifs discussed in Section 2.3.5 where a tetrameric cytochrome *cb*_562_ was assembled by complexation of Zn^2+^ to the PB (Fig. 19).^229^ Tezcan *et al.* investigated extensively the assembly of such PNs based on metal–ligand SLs^127^,^234^ and studied the effect of different amino acid mutations on the antimicrobial effect to derive the optimal cytochrome *cb*_562_ mutant. They next implemented assembly in *Escherichia coli* cells by including an N-terminal leader sequence in the cytochrome *cb*_562_ mutant in order to translocate them to and mature in the periplasm of the cells and performed the protein expression in LB media supplemented with 50 μM of ZnCl_2_.^229^ The localization in the periplasm is instrumental for the formation of disulfide bridge between the monomers for subsequent binding to Zn^2+^. The periplasmic contents were extracted, and SEC showed ∼70% tetramer formation. The *in vivo* β-lactamase activity of the PN was evaluated and it was shown that it is functional in the periplasm and allow the *Escherichia coli* cells to grow with ampicillin (a β-lactam antibacterial chemotherapeutic drug) concentrations in the range of 0.8 to 1.1 mg L^–1^, in contrast to a negative control.^229^

As shown in the examples discussed in this section, there is a rapid development in devising functional PNs for various biological applications, ranging from simple biocatalysis, to biotemplating, drug/protein delivery, and even assembly of bioactive PNs *in vivo* to control cellular functions, which is an exciting prospect for the further development in this field.

## 4. Conclusions

Nature has produced a refined machinery to generate and assemble precise PNs in a spatially defined fashion. The cell controls formation and degradation of PN in response to a multitude of stimuli and with fine-tuned stabilities. Uncovering and transferring such strategies using synthetic tools has proven challenging at first, although remarkable results have already been presented. In the past decades, with the increased understanding of protein structures, the development in chemical and biological research to emulate Nature's toolbox has advanced significantly. The major features that are essential for constructing PNs have been highlighted throughout this review. In summary, the preparation of the customized protein building blocks, the selection of the supramolecular recognition units, and the chemical design of the interconnecting linkers played an essential role in the resultant morphology, stability and properties of the final PNs. The levels of complexity of the PNs are steadily increasing with the development of more sophisticated chemical tools such as site-directed protein modification and, when applied in combination with genetic tools, gives rise to a greater versatility and diversity in protein structures and functions.

In this regard, the available chemical toolbox for the engineering of supramolecular protein nanostructures has certainly matured and, in most cases, the correlation of the different building blocks and structure formation can be predesigned to achieve functional PNs able to address various biological challenges, which has seen an exponential expansion since the early 2000s. The growth in the field has certainly been aided by the parallel development of high resolution analytical tools such as TEM and AFM, which allow better characterization and understanding of formed nanostructures as well as the building block-structure–function relationships. At the same time, more sophisticated biophysical techniques have also become available that allows for quantification of PB–PB and PB–SL interactions.

Non-covalent or covalent assembly have remained the chemical tools of choice. PNs formed in this manner could be transient in terms of stability for the former approach or only susceptible to proteolytic cleavage in the latter. As a perspective, it could be attractive to adopt new chemical tools such as dynamic covalent bonds including C–N,^235^–^237^ S–S^237^,^238^ and B–O^239^ bonds, which exhibit higher bond stability compared to non-covalent interactions but also provide reversibility. Such interactions could see a transition to dynamic PNs that allow predefined switching between structures and properties and at the same time, possess high stability due to the nature of the covalent bonds. Although site-selective chemical modification for mono- and dual-functionalization of proteins are well-established, it has not been employed extensively to design PB and this should be delved into to enlarge the combinatorial library for PN formation.

In addition, the implementation of PNs in biomedicine would require profound knowledge of their interactions with cells, which is still in the infancy stage and needs to be further developed. Such studies would also hold immense potential in synthetic biology, for the assembly of PNs *in vivo* to engineer artificial cellular components that can completely reprogram cellular functions. The chemical toolbox presented herein serves as basis, but additional features need to be implemented to construct PNs with exciting features, which Nature and biotechnology alone cannot achieve to bring the field forward and to provide new avenues in biomedicine and materials design.

## Conflicts of interest

There are no conflicts to declare.
